# The trapping of live neutrophils by macrophages during infection

**DOI:** 10.1038/s41419-025-07808-5

**Published:** 2025-07-03

**Authors:** Kelley N. Cooper, Marina Terekhova, Barbara Potempa, Ana D’Aubeterre, Jerome Timbol, Si An Chen, Antoine Dufour, Shunying Jin, Maxim N. Artyomov, Jan Potempa, Juhi Bagaitkar

**Affiliations:** 1 Center for Microbe and Immunity Research, Nationwide Children’s Hospital, Columbus, OH USA; 2 Department of Pathology and Immunology, Washington University School of Medicine in St. Louis, St. Louis, MO USA; 3 Department of Oral Immunology and Infectious Diseases, University of Louisville, Louisville, KY USA; 4 Department of Physiology & Pharmacology, Cumming School of Medicine, University of Calgary, Calgary, AB Canada; 5 Snyder Institute for Chronic Diseases, Cumming School of Medicine, University of Calgary, Calgary, AB Canada; 6 Department of Microbiology, Faculty of Biochemistry, Biophysics and Biotechnology, Jagiellonian University, Kraków, Poland; 7 Department of Pediatrics, The Ohio State University College of Medicine, Columbus, OH USA

**Keywords:** Inflammation, Immune evasion

## Abstract

Neutrophils are highly abundant in the oral mucosal tissues, and their balanced activation and clearance are essential for immune homeostasis. Here, we demonstrate that neutrophils infected with the bacterial pathogen *Porphyromonas gingivalis* (*Pg*) are captured alive by macrophages in a manner that bypasses all known receptor-ligand interactions involved in the phagocytosis of either live or dead cells. Mechanistically, upon interaction with *Pg*, or its protease RgpB (gingipains), live neutrophils undergo rapid remodeling of their proteomes, generating neoepitopes. N-terminomics-based proteomic profiling identified multiple RgpB cleavage sites on several azurophilic granule proteins that are translocated to the surface of live neutrophils via low-level degranulation and activate macrophage α_M_β_2_ integrin receptors, thus mediating internalization of non-apoptotic neutrophils within macrophage phagosomes. Macrophages with entrapped live neutrophils exhibit phenotypic and transcriptional reprogramming, consistent with inflammatory outcomes in vitro and in vivo. In contrast to the immunosuppressive outcomes associated with efferocytosis of apoptotic neutrophils, live neutrophil entrapment failed to fully activate several catabolic and metabolic processes and exhibited a defective activation of PPAR-γ mediated pro-resolution pathways, thereby promoting bacterial persistence and hindering the resolution of inflammation. Thus, our data demonstrate a novel immune subversion strategy unique to *Pg* and reveal a previously unknown mode of live neutrophil sequestration into macrophages during an infection.

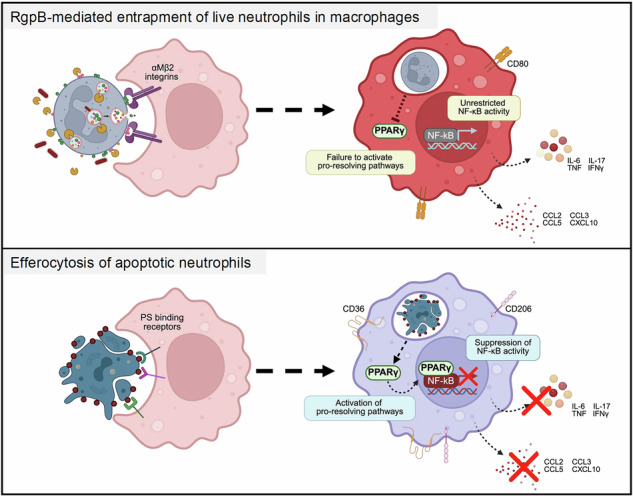

## Introduction

Neutrophils are the most abundant leukocytes in the body and play important roles in immune surveillance, antimicrobial immunity, and tissue homeostasis. Upon recruitment to sites of infection, they rapidly phagocytose and kill invading bacteria by degranulating and releasing antimicrobial mediators, thus playing a critical role in sterilizing immunity. Most recruited neutrophils eventually undergo apoptosis, a process characterized by distinct molecular alterations to the cell surface proteins, sugars, receptor repertoire, and plasma membrane lipid composition that collectively serve as “eat me” signals and facilitate their clearance via efferocytosis within tissues. For example, the exofacial localization of phosphatidylserine (PS) is a well-conserved, apoptosis-associated signal recognized directly or indirectly by many efferocytic receptors that regulate the phagocytic uptake of dying neutrophils within macrophages, termed efferocytosis [1, 2]. While most infected, aged, or apoptotic neutrophils (ANs) are cleared via efferocytosis [3] or phagoptosis [4], several studies now show that the expression of apoptotic markers does not always predicate neutrophil engulfment by another cell, and under certain circumstances, live neutrophils might also be internalized by other live cells [5–8].

Viable neutrophils appear to invade and transit through endothelial cells during transcellular migration by forming a transient transcellular channel between the apical and basal membranes of endothelial cells within blood vessels to exit circulation [6]. Emperipolesis is another example in which live neutrophils invade megakaryocytes and either remain within vacuolar structures called ‘emperisomes’ or enter the megakaryocyte cytosol, eventually egressing and carrying the megakaryocyte membrane [5, 7]. Transcellular migration and emperipolesis is mediated in part via ICAM-1 and β_2_ integrin interactions, transcellular cup formation, and actin remodeling [5, 6]. Recently, using a model of allergic inflammation, Mihlan et al. showed that live neutrophils can also be entrapped by degranulating mast cells (MCs) in a leukotriene B4 (LTB4) dependent manner, resulting in a cell-in-cell structure [8]. Here, we show that live neutrophil ingestion can also occur under pathogenic conditions, such as bacterial infections, creating an environment conducive for bacterial survival.

*Porphyromonas gingivalis* (*Pg)* is a human-adapted bacterial colonizer of the oral mucosal tissues and is etiologically associated with periodontal inflammation and other chronic inflammatory or degenerative diseases [9]. Its pathogenic potential is primarily linked to the production of arginine-specific (HRgpA and RgpB) and lysine-specific (Kgp) proteases called gingipains that cleave proteins at the arginine (Arg-X-aa) and lysine (Lys-Xaa) peptide bonds, respectively [10]. Gingipains cleave a large number of host proteins and are particularly adept at manipulating neutrophil antimicrobial and inflammatory responses [1, 11, 12]. Interestingly, despite nearly identical catalytic activity, HRgpA and RgpB differentirely impact neutrophil responses and viability. While HRgpA induces robust NETosis and apoptosis, RgpB minimally impacts neutrophil viability or lifecycle, but instead dampens their antimicrobial capacity and induces the cleavage of non-phagocytic signals (CD31) of neutrophil surface macrophages [11, 13, 14]. However, the full extent of RgpB-mediated modifications to the neutrophil proteome and its impact on tissue inflammatory responses or *Pg* fitness with the host have not been explored. Here, we show that through the activity of RgpB, *Pg* remodels the proteomes of live neutrophils, generating non-canonical uptake signals that engage α_M_β_2_ integrin receptors on macrophages and their subsequent sequestration within macrophages. The entrapment of live neutrophils within macrophages dysregulates pro-resolution pathways and favors bacterial persistence in vivo.

## RESULTS

### RgpB protease is necessary and sufficient for the entrapment of live neutrophils within macrophages

We previously showed that purified RgpB can cause a modest cleavage of antiphagocytic signals on the surface of live neutrophils, causing their ingestion by a subset of human monocyte-derived macrophages [14]. However, since *Pg* produces several virulence factors, we first determined whether gingipains alone were necessary and sufficient for the entrapment of live neutrophils upon infection. Human neutrophils infected with *Pg* mutants lacking either the major fimbriae (*ΔfimA*), peptidylarginine deaminase (*Δppad*), or gingipains (*ΔrgpA, Δrgpb*, and *Δkgp* triple mutant, abbreviated as *ΔKRAB*) for 1 h did not externalize PS (Fig. S1A), an ‘eat me signal’ that mediates efferocytosis or phagocytosis of dying and distressed cells by a large number of efferocytic receptors. Next, we measured whether macrophages ingested *Pg*-infected neutrophils despite the lack of any obvious “eat me” signals using well-established in vitro and in vivo efferocytic assays [15, 16].

Murine peritoneal exudate macrophages (PEMs) were co-cultured with neutrophils and uptake was determined by histochemical staining for myeloperoxidase (MPO), which is highly expressed in neutrophils and absent in macrophages [15] (Fig. S1B). Despite the absence of PS, neutrophils infected with *Pg* (WT, *ΔfimA*, and *Δppad*) were rapidly internalized by macrophages with efficiency comparable to the uptake of PS-expressing ANs. Interestingly, the phagocytosis of *ΔKRAB*-infected neutrophils was significantly dampened, indicating that gingipain-mediated proteolytic processing was essential for the sequestration of live neutrophils within macrophages (Fig. S1B, C). RgpB, unlike HRgpA, does not induce apoptosis or NETosis in neutrophils upon prolonged treatment but instead facilitates their live uptake by macrophages [13, 14]. Selective inhibition of RgpB activity on both live *Pg* and purified RgpB enzyme using a highly specific non-reversible inhibitor, D-Phe-Phe-Arg-chloromethylketone (FFR-CMK), significantly dampened uptake (Fig. S1D, E), confirming that the proteolytic activity of RgpB was necessary and sufficient for the entrapment of live *Pg*-infected neutrophils within macrophages. While the gingipain deficient strains were instrumental in understanding the role of gingipains in live neutrophil entrapment, they are rapidly cleared in vivo, making direct comparisons of RgpB mutant and WT strains improbable in vivo. Thus, we worked with purified RgpB for the rest of our experiments and confirmed key findings with *Pg* infected neutrophils. This approach is biologically relevant as gingipains are actively secreted and found at sites distant from the oral cavity, such as joints, brain, liver, lungs, and blood in humans [17].

**Fig. 1:**
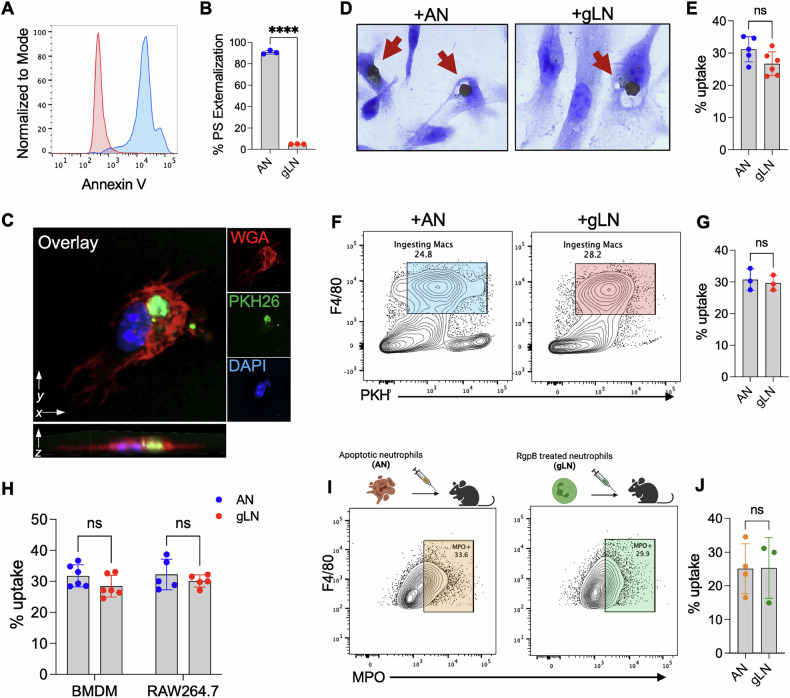
Purified RgpB mediates entrapment of live neutrophils. **A**, **B** Purified human neutrophils were incubated with *P. gingivalis* protease RgpB (300 nM) for 1 h or with cycloheximide (50 μg/mL) for 18 h to induce apoptosis. After treatments, phosphatidylserine (PS) externalization was assessed by Annexin V staining of apoptotic neutrophils (ANs; blue histogram) or RgpB-treated live neutrophils (gLNs; red histograms) from 3 independent donors. Relative uptake of ANs and gLNs by various macrophage types was determined in vitro and in vivo by the following assays: **C** The phagocytosis of PKH26-green labeled gLNs by murine peritoneal exudate macrophages (PEM) after 2 h of co-incubation was determined by confocal microscopy. PEM membranes were labeled with wheat germ agglutinin (red). **D**, **E** To quantitatively assess relative uptake rates in vitro, ANs or gLNs were incubated with PEMs for 2 h, and uningested neutrophils were removed by washing with PBS. Phagocytosing macrophages were determined by histochemical staining for myeloperoxidase (MPO), a protein selectively expressed in neutrophils. **D** Representative images of MPO^+^ PEMs at 100x magnification are shown. **E** % uptake was determined by counting phagocytosing or MPO^+^ (brown staining) PEMs and expressed as a percentage value over total PEMs. At least 300 macrophages were counted (blinded) for each replicate, and data from 3 independent experiments is shownas mean ± SD, and data points indicate biological replicates; ****p < 0.0001 (unpaired *t*-test). **F**, **G** For flow-based in vitro quantification of phagocytosis, PEMs were incubated with PKH26-labeled human gLN or AN. Macrophages with attached or incompletely ingested neutrophils were excluded by gating out (hCD45^+^ F4/80^+^, PKH26^+^). The relative abundance of phagocytosing macrophages (F4/80^+^, PKH26^+^) is shown as mean ± SD, and data points indicate biological replicates. Statistical significance was calculated using an unpaired *t*-test. **H** The relative phagocytic rates of ANs or gLNs were also determined in murine bone marrow-derived macrophages (BMDM) and RAW264.7 macrophages by MPO-based phagocytosis assay described in (**D**, **E**). Averaged data (mean ± SD) from three independent experiments is shown. Data points indicate biological replicates and statistical significance was calculated by two-way ANOVA and Šídák correction (**I**, **J**). For in vivo uptake assay, wildtype (WT) mice were injected intraperitoneally (i.p.) with 10^7^ human neutrophils (AN or gLN). 4 h after injection, the peritoneal cells were collected by lavage, and phagocytosing macrophages (hCD45^-^, F4/80^+^, hMPO^+^ population) were determined by flow cytometry. Data are shown as mean ± SD, and data points indicate biological replicates. The illustration above, data panels in (**I**), was createdusing Biorender.com.

We developed two separate model systems to track neutrophil entrapment and its role in modulating macrophage responses in vitro and in vivo. The dual-species model, where human neutrophils are fed to murine peritoneal macrophages (PEMs)[15, 16], allows for the use of species-specific reagents to exclude contaminating signals (passenger transcripts, proteins, cellular markers) from internalized live or apoptotic cells and was used to delineate macrophage transcriptional response to live neutrophil entrapment. This approach was complemented by using a physiologically relevant single-species model (murine neutrophils were fed to murine macrophages) in select experiments. Similar to infection with live bacteria, human neutrophils exposed to purified RgpB (gLN) did not induce PS expression (Fig. 1A, B) and were rapidly internalized into early phagocytic compartments by PEMs (Fig. 1C). The efficiency of uptake was comparable to that of ANs as determined by two independent in vitro efferocytosis assays (Fig. 1D–G). Of note, MPO histochemical staining and flow-based uptake assays were highly concordant and showed ~30% uptake of gLNs and ANs within 2 h of co-culture with macrophages.

To rule out the impact of tissue origin or priming status of macrophages in live neutrophil entrapment, we co-cultured gLNs and ANs with naïve bone marrow-derived macrophages (BMDMs) and the RAW264.7 macrophage cell line. Our data show comparable uptake rates of gLN and ANs in these cell types (Fig.1H). We injected human gLNs or ANs into the inflamed peritoneal cavities of mice and determined uptake by flow cytometry using a human-specific anti-MPO antibody. Concordant with our in vitro data, gLN entrapment was also observed in vivo and occurred with the same efficiency as the uptake of ANs (Fig. 1I, J). Similar uptake rates were also observed in the single-species model, where gingipain-treated murine bone marrow neutrophils (g-BMN) were entrapped in vitro and in vivo at similar efficiency as apoptotic murine neutrophils, confirming that our observations are independent of differences in the species of origin (Fig. S2). We also if determined RgpB exposure induced the uptake of other cell types. PEMs were co-cultured with PKH-labeled apoptotic or RgpB-treated live thymocytes (apop-thym and gL-thym, respectively) and % uptake determined by flow cytometry. Interestingly, we observed that gL-thymocytes (Fig. S3A–D) were not internalized by macrophages, suggesting that RgpB was selectively modifying neutrophil-specific protein(s), which might be essential for live neutrophil entrapment. Altogether, these data show that *Pg*, relies on the proteolytic activity of RgpB to mediate the entrapment of live neutrophils within macrophages.

### RgpB cleaves and mobilizes neutrophil granule proteins to the surface

**Fig. 2:**
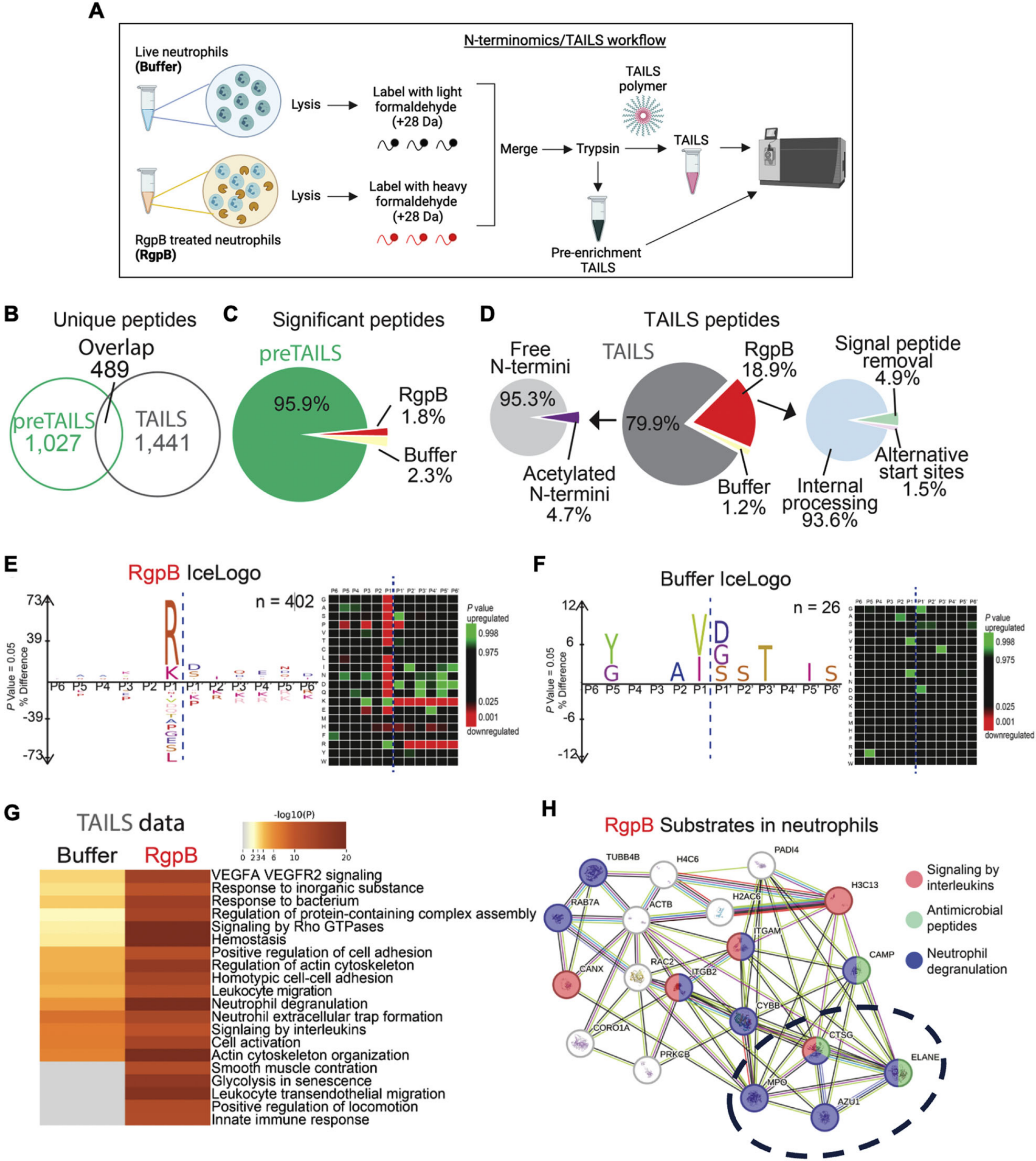
RgpB proteolytically modifies the intracellular neutrophil proteome. Live human neutrophils were incubated with active 300 nM RgpB (gLN) for buffer alone (Buffer) and subject to terminal amine isotopic labeling of substrates (TAILS) mass spectrometry (TAILS-MS). **A** Schematic depicting TAILS-MS workflow (generated using Biorender.com). Briefly, after blocking primary amines (not shown), samples underwent isotopic labeling with heavy (deuterated) or light formaldehyde and digestion with trypsin. After trypsin digestion, a fraction of each sample was subject to pre-enrichment TAILS (shotgun analysis or pre-TAILS). The rest underwent removal of tryptic N-terminal peptides using a high molecular weight dendritic polyglycerol aldehyde polymer, leaving the naturally blocked or labeled mature and neo-N-termini unbound via negative selection (flow-through). TAILS peptides were recovered by size exclusion filtration and analyzed by liquid chromatography coupled with tandem mass spectrometry (LC-MS/MS). **B** The numbers of unique and shared peptides between TAILS and preTAILS analysis are shown. **C** The numbers of statistically changing peptides using an interquartile boxplot analysis between the pre-TAILS samples are shown. **D** Left: Distribution of N-terminal peptides in the TAILS enrichment. Middle, statistically changing peptides using an interquartile boxplot analysis. *Right*, Distribution of post-translational peptide modifications as analyzed using TopFINDER. For a complete list of peptides in figures (**B**, **C**), also see Supplementary Table 1. **E** *Left*, peptide sequence profiles of significantly elevated neo-N-terminal peptides in RgpB treated neutrophils identified in the TAILS analysis using IceLogo. *Right*, Cleavage sites identified as RgpB-treated neutrophils are depicted as heatmaps from P6 to P6′ residues. Green: Upregulated. Red: Downregulated. **F** *Left*, peptide sequence profiles of significantly elevated neo-N-terminal peptides in untreated (buffer) neutrophils identified in the TAILS analysis using IceLogo. Significantly (*p* < 0.05) overrepresented amino acids are shown above the x-axis, while the underrepresented residues are shown below the x-axis. Statistical analysis was determined by a two-tailed unpaired Student’s *t*-test and adjusted for multiple comparisons. **G** Metascape analysis of the TAILS data of different pathways between buffer- and RgpB-treated neutrophils is shown. **H** STRING-db analysis of RgpB cleaved substrates is shown. An enrichment was detected for neutrophil degranulation (blue), signaling by interleukins (red) and antimicrobial peptides (green). The dotted circle shows an enrichment of neutrophil azurophilic granule proteins (cathepsin G (CTSG); neutrophil elastase (ELANE); myeloperoxidase (MPO) and azurocidin (AZU1)) as RgpB substrates.

Since RgpB’s proteolytic activity was central to the entrapment of live neutrophils (Fig. S1), we hypothesized that RgpB facilitated the generation of neo-epitopes that engaged macrophage phagocytic receptors, to drive live neutrophil phagocytosis. To identify RgpB protein substrates within live neutrophils, we resorted to the terminal amine isotopic labeling of substrates (TAILs) approach, followed by liquid chromatography and tandem mass spectrometry (LC-MS/MS) based identification. TAILS is a quantitative proteomics approach that facilitates the unbiased identification of protease substrates and cleavage sites in biological samples [18]. In this approach, all naturally occurring N termini and neo-N-termini generated by RgpB are blocked by differential isotopic labeling. Briefly, neutrophils from control and RgpB exposed samples were isotopically labeled with light formaldehyde (+28 Da dimethylation) or heavy formaldehyde (+34 Da dimethylation), respectively. Pooled samples were then trypsinized to reduce complexity, and then N-termini were enriched by incubation with dendritic polyglycerol aldehyde TAILS polymer that removes unlabeled proteins as previously described in [19] and illustrated in (Fig. 2A). The global proteomes were compared by a shotgun (pre-enrichment TAILs) proteomic analysis, while TAILS focused on the proteomic analysis of N-termini in all proteins [18, 20]. After sample acquisition and LC-MS/MS analysis, data were analyzed using MaxQuant [21] at 1% FDR.

Shotgun (pre-TAILS) analysis of RgpB treated or untreated neutrophils from 3 donors yielded 1441 unique peptides, and the TAILS analysis yielded 1930 unique peptides where 489 were identical to the pre-enrichment TAILS analysis (Supplementary Table 1; Fig. 2B). In the pre-TAILS data, we identified a significant change of 1.8% of peptides in the RgpB treated neutrophils and 2.3% in the untreated control (Fig. 2C). Next, we analyzed the N-terminal processing in the RgpB-treated samples. Protease-generated neo-N termini should only be present in the protease-exposed samples, and not in the untreated (buffer control) samples. Our TAILS analysis identified predominantly internal N-termini (93.6%), in addition to other proteoforms, including N-termini generated by signal peptide removal (4.9%) and alternative start sites (1.5%) (Fig. 2D). Next, we generated IceLogos to determine cleavage site preferences between RgpB treated and untreated (buffer) neutrophils (Fig. 2E, F). RgpB cleaves proteins at the C-terminal side of arginine residues or the Arg-X-aa bonds [10]. Thus, as expected, we identified a preference for P1 arginine residues and a preference for aspartic acid and serine in P1’ in the RgpB-treated group (Fig. 2E). Pathway analysis ranked several RgpB-cleaved proteins within the neutrophil transmigration, actin cytoskeletal organization, antimicrobial defense, and degranulation pathways (Fig. 2G). Using Metascape, we generated a pathway enrichment analysis network by merging all significantly changed proteins and determined their interactions across pathways using STRING db analysis (Fig. 2H; Supplementary Tables 1, 2).

### Exocytosed and proteolytically inactive MPO and NE are ligands for β_2_ integrin-mediated entrapment of live neutrophils within macrophages during Pg infection

**Fig. 3:**
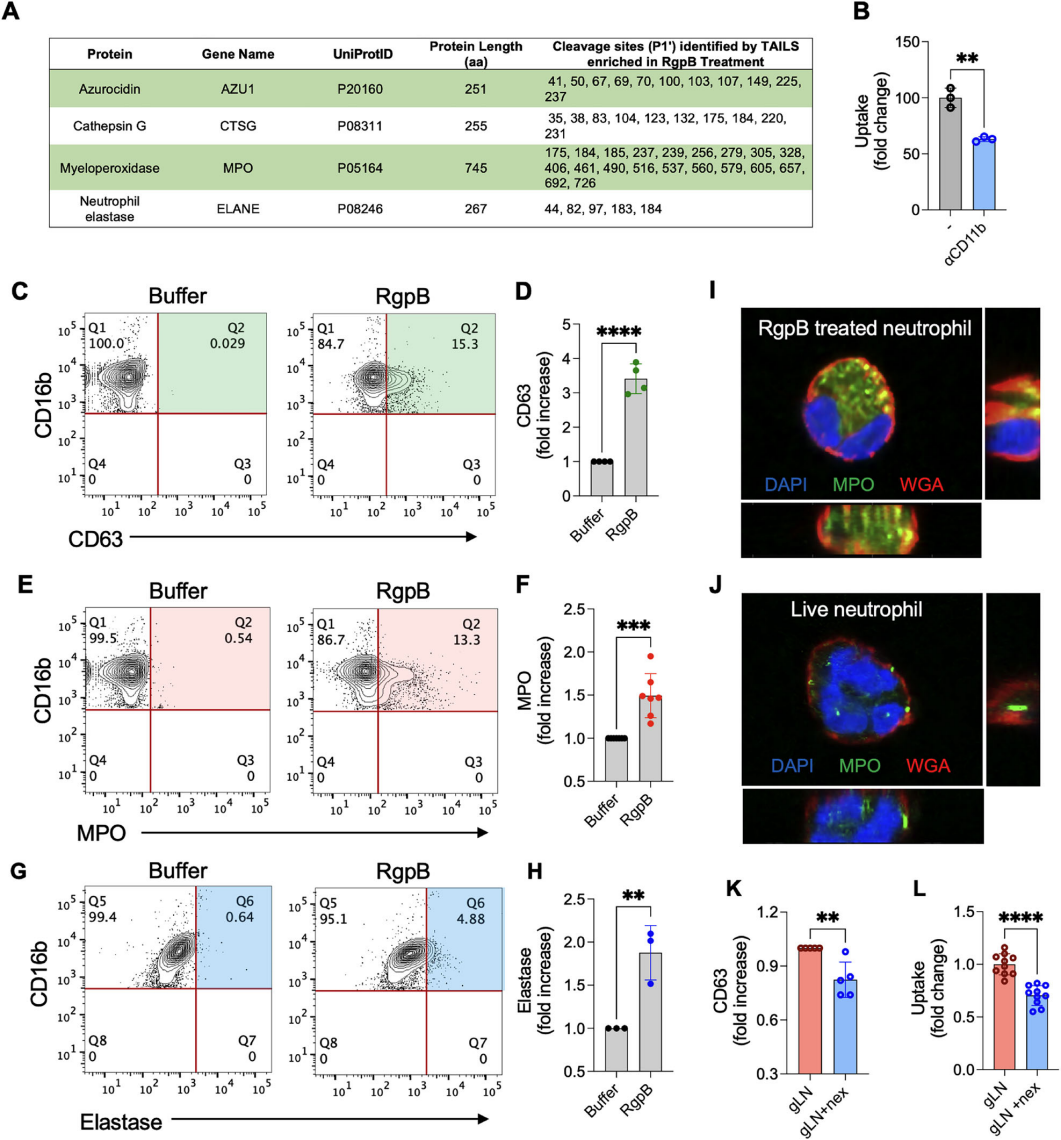
RgpB generates novel uptake ligands from neutrophil granule proteins. **A** RgpB cleavage sites on neutrophil granule proteins (identified by TAILS-MS) that are shown. **B** Peritoneal exudate macrophages (PEMs) were co-cultured with RgpB-treated live neutrophils (gLNs) in the presence of absence of neutralizing antibodies to αmβ_2_ receptors (anti-CD11b) for 1 h and phagocytosing PEMs were scored based on MPO positivity, as described in Fig. 1. Data (mean ± SD) from three biological replicates is shown as fold change over control. Statistical differences were measured by *t*-test; **p < 0.02. **C**–**H** Translocation of the azurophilic granule marker CD63 (**C**, **D**) and the azurophilic granule proteins MPO (**E**, **F**) and neutrophil elastase (**G**, **H**) to the cell surface of untreated (Buffer) or RgpB treated neutrophils (300 nM, 1 h) was determined by flow cytometry. CD16b was used to gate on human neutrophils. Representative contour plots are shown in (**C**, **E**, **G**) and quantification (**D, F, H**) of marker positive cells is shown as fold change over buffer control. Data point from 3 to 7 donors are shown as mean ± SD. Statistical significance was calculated using an unpaired *t*-test: **p < 0.02, ***p < 0.001, ****p < 0.0001. **I**, **J** The translocation of MPO to the surface of live neutrophils upon RgpB-treated by confocal microscopy is shown. **K** Neutrophils were treated with RgpB in the presence of 1 μM nexinhib20 (nex) to inhibit degranulation. Averaged data from 5 independent donors is shown as fold change in CD63 surface translocation by flow cytometry. **L** gLNs and gLNs+nex were co-cultured with PEMs, and uptake was determined by MPO staining and quantification of MPO^+^ PEMs. Data are shown as mean ± SD, and each data point represents a biological replicate. Statistical significance was calculated using an unpaired *t*-test; **p < 0.02.

In the TAILS dataset, many azurophilic granule proteins such as MPO, cathepsin G (CatG), neutrophil elastase (NE), proteinase 3 (PR3), and azurocidin that are almost exclusively expressed in neutrophils, were cleaved by RgpB at multiple arginine residues,consistent with the enrichment of the neutrophil degranulation pathway (Fig. 3A; Fig. 2H–G). This drove our interest as live RgpB-treated thymocytes that do not express neutrophil granules protein were not ingested by macrophages (Fig. S3A–D). Thus, we focused on neutrophil granule proteins modified by RgpB and determined whether they played a role in live neutrophil entrapment.

Upon release, neutrophil granule proteins can be sequestered on the exofacial membrane via covalent or ionic interactions [22–26]. Interestingly, neutrophil granule proteins such as MPO and NE have been shown to bind the α_M_β_2_ (CD11b/CD18) integrin receptor, an essential phagocytic receptor with over a hundred reported ligands [22–27]. α_M_β_2_ is a member of the β_2_ integrin family of receptors previously linked with orchestrating the internalization of live neutrophils during transendothelial migration and emperipolesis [5, 6]. We observed that gLN ingestion was significantly dampened in the presence of neutralizing anti-CD11b antibodies that block α_M_β_2_ (Fig. 3B). iC3b, the canonical ligand for α_M_β_2_-mediated uptake of apoptotic cells, was absent on gLNs (Fig. S3E, F) supporting a role for alternate α_M_β_2_ ligands in live neutrophil entrapment. We observed that RgpB treatment significantly increased the surface abundance of CD63 (Fig. 3C, D), a marker associated with the exocytosis of azurophilic granules [28] as well as the cell membrane association of MPO and NE on live neutrophils (Fig. 3E–J). Finally, to confirm that degranulation of azurophilic granules specifically was essential for gLN uptake, we incubated live neutrophils with nexinihib20, which blocks azurophilic granule exocytosis [29], during RgpB treatment. Nexinhinib20 significantly reduced the exocytosis of azurophilic granules which correlated with a significant drop in CD63 expression and consequently gLN uptake (Fig. 3K, L). These differences were not due to any off-target impact of nexinihib20 on RgpB activity (Fig. S3G). Thus, our data show that RgpB treatment induces low-level degranulation and opsonization of the neutrophil surface by inactive azurophilic granule proteins, facilitating α_M_β_2_ mediated live neutrophil entrapment in macrophages.

### The entrapment of live neutrophils within macrophages activates pro-inflammatory pathways

**Fig. 4:**
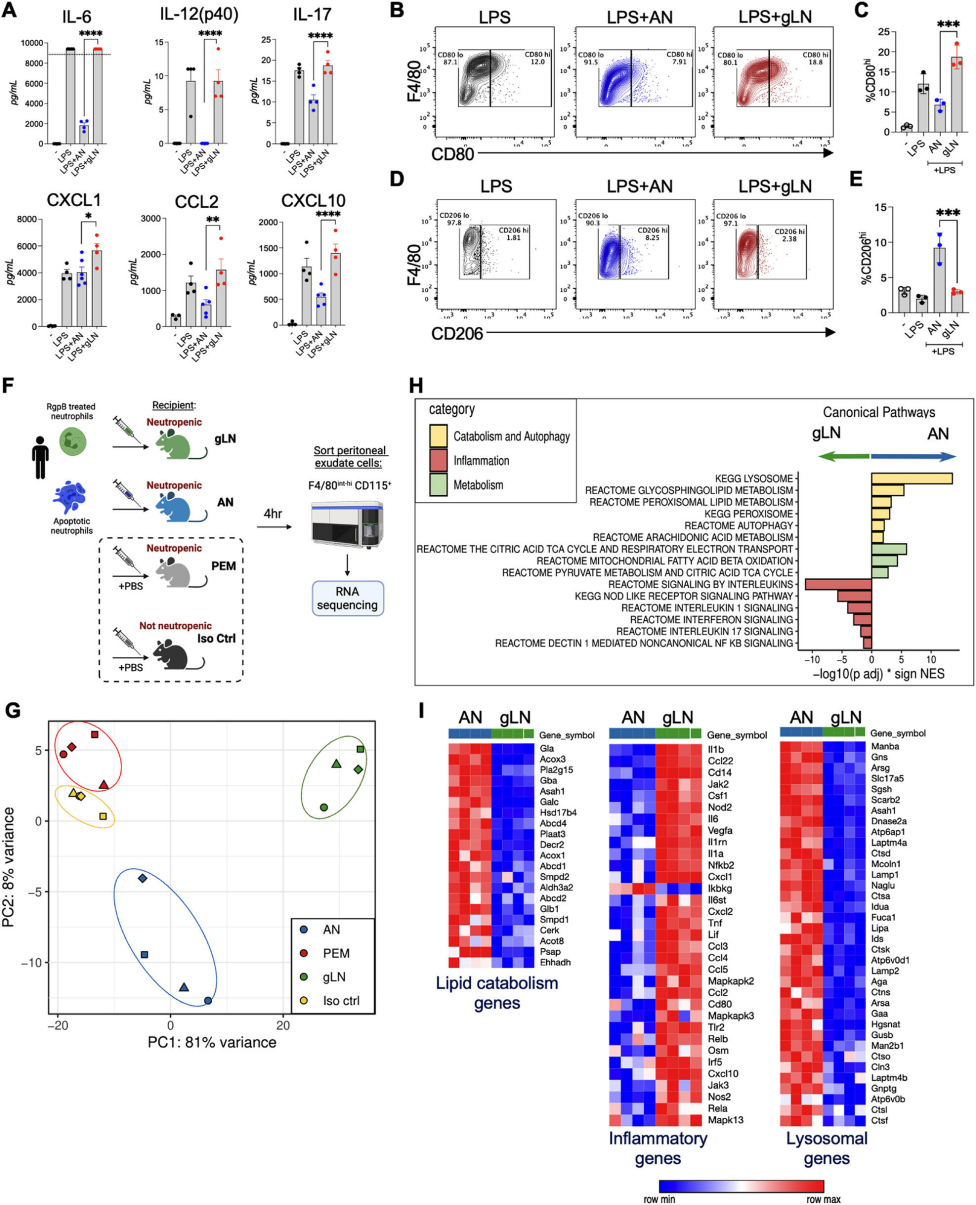
Entrapment of live neutrophils does not mediate phenotypic switching to pro-resolving state in vivo and in vitro. **A–E** Murine PEMs were co-cultured with human gLNs or ANs for 2 h, and uningested cells were removed before adding LPS (10 ng/ml) for 18 h. Inflammatory cytokine levels in cell-free supernatants were determined by 32-plex cytokine array (**A**), and the expression of pro-inflammatory (CD80) and pro-resolving (CD206) cell surface markers in AN or gLN ingesting PEMs was determined by flow cytometry (**B**–**E**). Relative shifts in cell surface marker expression is shown as contour plots in (**B**, **D**), and % shift quantified from 3 to 5 individual biological replicates in (**C** and **E**), respectively. Data are shown as mean ± SD and statistical significance calculated using one-way ANOVA with Tukey’s correction: *p < 0.05, **p < 0.01, ***p < 0.001, ****p < 0.0001. **F** Experimental design for dual-species RNA-seq created using Biorender.com: WT mice were injected intraperitoneally (i.p.) anti-Ly6G antibodies to experimentally induce neutropenia or isotype control antibodies for 3–5 days. Peritonitis was induced with an i.p. injection of 5 mM sodium periodate. 72 h later, 10^7^ human gLNs or ANs suspended in sterile PBS were injected i.p. into the inflamed peritoneal cavities of neutropenic WT mice. Controls included neutropenic mice that did not receive AN/gLN injections (denoted as PEM) and non-neutropenic mice (injected with isotype control antibodies) that did not receive AN/gLN injections (denoted as iso ctrl). After 4 h, mice were euthanized, and peritoneal macrophages were sorted using the gating strategy described in Fig. S5A for RNA-seq. **G** PCA plot depicting clustering of PEMs based on ingested neutrophils (AN or gLN), PEMs, and Iso ctrl. **H** Bar plots for enriched pathways comparing AN vs. gLN groups are shown. Bars to the left depict upregulated pathways in gLN ingesting PEMs, while bars to the right depict upregulated pathways in AN ingesting PEM. **I** Heatmaps of significant differentially expressed genes between macrophages that ingested AN vs. gLN. Red indicates highly expressed genes. Statistical significance can be found in Supplementary Tables 3, 4.

Next, we focused on the impact of live neutrophil entrapment on macrophage inflammatory responses. In other cases of internalization of live neutrophils, such as transendothelial migration and emperipolesis, there was a minimal impact on the inflammatory profiles of ingesting cells [30]. However, activated MCs that entrapped neutrophils showed considerable metabolic and proteomic changes that bolstered their immune function [8]. In contrast, efferocytosis of apoptotic cells predominantly activates immunosuppressive signaling and tolerance, or immunological refractoriness, to subsequent pathogenic insults [31, 32]. Signaling downstream of PS sensing receptors [16, 33, 34] and the catabolic breakdown of apoptotic cells within efferosomes [35] synergistically activate transcriptional, metabolic, and epigenetic programs that transform the efferocytosing macrophage from an inflammatory state (CD80^hi^) towards a pro-resolving (CD206^hi^) state, with an overall suppressed ability to produce pro-inflammatory cytokines [35–38]. Interestingly, we observed that compared to efferocytosis of ANs, gLN ingestion led to significantly higher production of pro-inflammatory cytokines and chemokines (Fig. 4A; Fig. S4A) and macrophage polarization towards a pro-inflammatory state (Fig. 4B–E). *Pg* infected live neutrophils phenocopied our observations with gLNs (Fig. S4B–D). Thus, unlike apoptotic cells previously shown to transcriptionally and epigenetically silence macrophages [38], the engulfment of viable neutrophils after RgpB treatment on *Pg* infection led to inflammatory outcomes.

To determine the impact of gLN entrapping in vivo, we adoptively transferred 10^7^ human neutrophils (gLNs or ANs) i.p into the inflamed peritoneal cavities of mice and flow-sorted peritoneal macrophages after 4 h for RNA-seq as illustrated in Fig. 4F (see Fig. S5A for sorting strategy). The dual-species approach allowed us to precisely track macrophage responses by aligning mouse-specific transcripts while excluding passenger transcripts (human origin) emanating from ingested targets (gLNs or ANs). To further increase the stringency of this approach, the recipient mice were made neutropenic before peritonitis induction [39, 40] to eliminate competition from endogenous neutrophils and allow for maximal uptake of injected gLNs/ANs. Principal component analysis showed that the depletion of neutrophils did not significantly impact macrophage transcriptomes, as seen by the close clustering of PEMs (anti-Ly6G injected) vs. isotype control mice (Fig. 4G). Strikingly, we saw significant differences in the transcriptomes of macrophages isolated from mice that were adoptively transferred with gLNs compared to mice that received AN (Fig. 4H, I). Consistent with published literature, we saw that the efferocytosis of apoptotic cells (ANs) induced a broad dampening of inflammatory pathways and activated genes in the metabolic and autophagy pathways essential for the catabolic breakdown of ingested efferosomal cargo (Fig. 4H, I; Supplementary Tables 1–4). In contrast, macrophages with entrapped live neutrophils upregulated multiple inflammatory pathways and showed a deficient activation of genes associated with lysosomal biogenesis and lipid catabolism (Fig. 4H, I; Supplementary Tables 3–6).

**Fig. 5:**
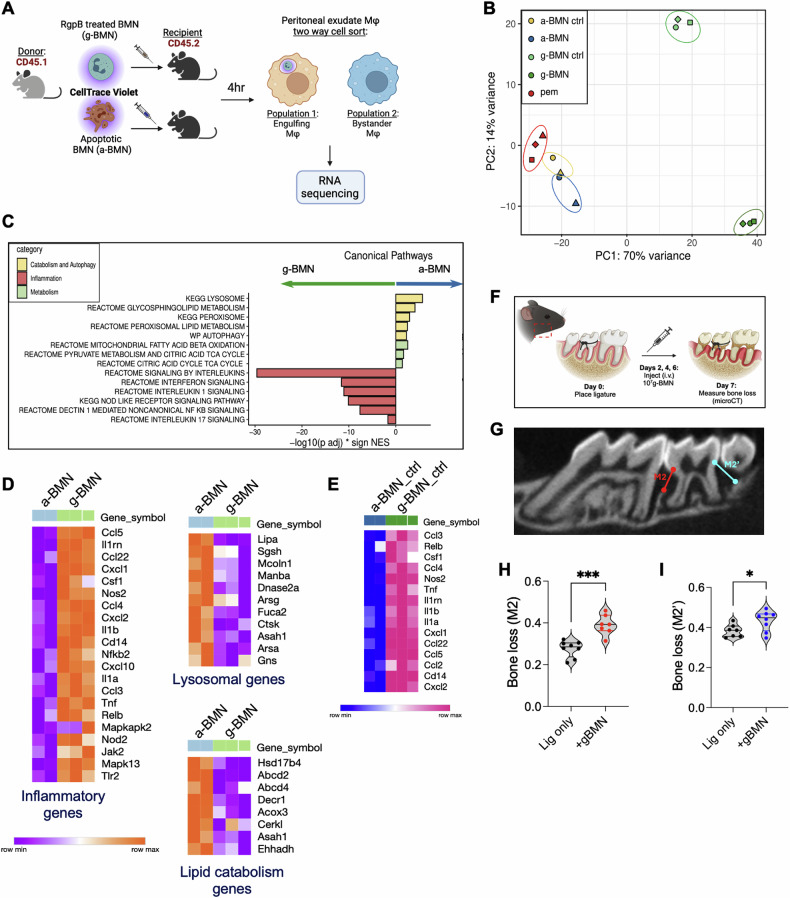
Live neutrophil trapping disrupts anti-inflammatory signaling and influences the tissue milieu. **A** Schematic of the experimental design was created using Biorender.com: Peritonitis was induced in WT mice with an i.p. injection of 5 mM sodium metaperiodate. 72 h after the onset of peritonitis, 10^7^ apoptotic or RgpB-treated murine bone marrow neutrophils (a-BMN and g-BMN, respectively) from CD45.1 donor mice were labeled with CellTrace Violet and injected into the inflamed peritoneal cavities of CD45.2 recipient mice. 4 h post-injection, peritoneal exudate macrophages (PEMs) were sorted into two populations of phagocytosing macrophages or ‘bystander’ macrophages based on Cell trace positivity using the gating strategy described in Fig. S5B. Sorted cells were analyzed by RNA-seq. **B** PCA plot depicting clustering of PEMs based on transcriptional responses of peritonitis mice that did not receive g-BMN or a-BMN injection (PEM), g-BMN or a-BMN ingesting macrophages, or ‘bystander’ macrophages from g-BMN or a-BMN injected mice (g-BMN ctrl and a-BMN ctrl, respectively). **C** Bar plot showing enriched catabolism & autophagy, inflammation, and metabolism pathways. Bars to the left depict upregulated pathways in g-BMN ingesting macrophages; bars to the right depict upregulated pathways in macrophages that ingested a-BMNs. Statistical significance can be found in. **D**, **E** Heatmaps showing significant differentially expressed inflammatory genes, lysosomal genes, and lipid catabolism genes in g-BMN vs a-BMN ingesting macrophages (**D**) or control non-effercytosing or bystander macrophages from same mice (**E**). Statistical significance amongst represented genes can be found in Supplementary Tables 7, 9. **F** Heatmap depicting significant differentially expressed inflammatory genes in a-BMN ctrl vs g-BMN ctrl macrophages. Pink indicates highly expressed genes. Statistical significance amongst represented genes can be found in Supplementary Table 8. Oral inflammation was induced in mice by placing ligatures on the second maxillary molars of wildtype mice as depicted in (**F**). On alternate days, mice received sterile saline (denoted as ‘Lig only’) or 10^7^ g-BMN via tail vein injection (i.v.). On day 7, mice were euthanized, and alveolar bone loss was assessed by μCT. **G** Representative μCT images showing alveolar bone recession surrounding ligature sites M2 and M2’ molars. **H**, **I** Quantification of M2 and M2’ bone loss is shown in mm. Averaged data from 7-8 mice is shown as mean ± SD, and statistical significance determined using an unpaired *t*-test; *p < 0.05, **p < 0.02.

To show that the ingestion of gLN vs AN impacted the transcriptome of the ingesting macrophage in a cell-intrinsic manner and was unrelated to species differences, we confirmed our findings in an allogenic or ‘same species’ model of efferocytosis. Bone marrow neutrophils (BMNs) were isolated from CD45.1 donor mice and fluorescently labeled with a tracer dye (CellTrace Violet) before RgpB treatment (g-BMN) or the induction of apoptosis (a-BMN), then injected into the inflamed peritoneal cavities of CD45.2 recipient mice (Fig. 5A). After 4 h, we used a 2-way sorting strategy to isolate ingesting and non-ingesting or ‘control’ or bystander macrophages from the peritoneal cavity based on CellTrace Violet positivity (see sorting strategy in Fig. S5B), followed by RNA-seq. This allowed us to determine the transcriptional responses to the phagocytic uptake of live vs. ANs in a cell-intrinsic manner. Consistent with our observations in the dual species model, we saw that the uptake of apoptotic or a-BMNs suppressed inflammatory pathways while live neutrophil entrapment (g-BMN) led to a significant upregulation of pro-inflammatory genes in both ingesting macrophages (Fig. 5B–D; Supplementary Tables 7–12). Thus, our data show a high concordance between our single species and dual species models. We also did RNA-seq on bystander macrophages from the peritoneal milieu that did not ingest neutrophils (referred to as a-BMN ctrl or g-BMN ctrl) to determine their transcriptional responses and observed distinct transcriptional profiles and clustering of bystander macrophages on PCA plots (Fig. 5B). Interestingly, the bystander macrophages from g-BMN injected mice showed an upregulation of inflammatory cytokine and chemokine genes (Fig. 5E; Supplementary Tables 7–12), indicating a reactive response to either the injection of gingipain treated BMNs or their uptake.

Finally, we used a ligature-induced periodontitis (LIP) model to determine the impact of gLNs in oral inflammation, a niche that *Pg* colonizes. Ligature placement in mice causes localized tissue trauma and recruitment of neutrophils to the oral soft tissues and gingiva. It also allows for the accumulation of oral microbes around the ligatures, all of which contribute to aggravated tissue injury and alveolar bone recession, as previously described [41]. Repeated injection of gLNs in LIP mice exacerbated oral inflammation and significantly higher alveolar bone recession (Fig. 5F–I), indicating that RgpB-induced modulation of live neutrophils worsened inflammatory outcomes in vivo.

### Inefficient activation of PPAR-γ signaling sustains NF-κB-mediated inflammation during live neutrophil entrapment

**Fig. 6:**
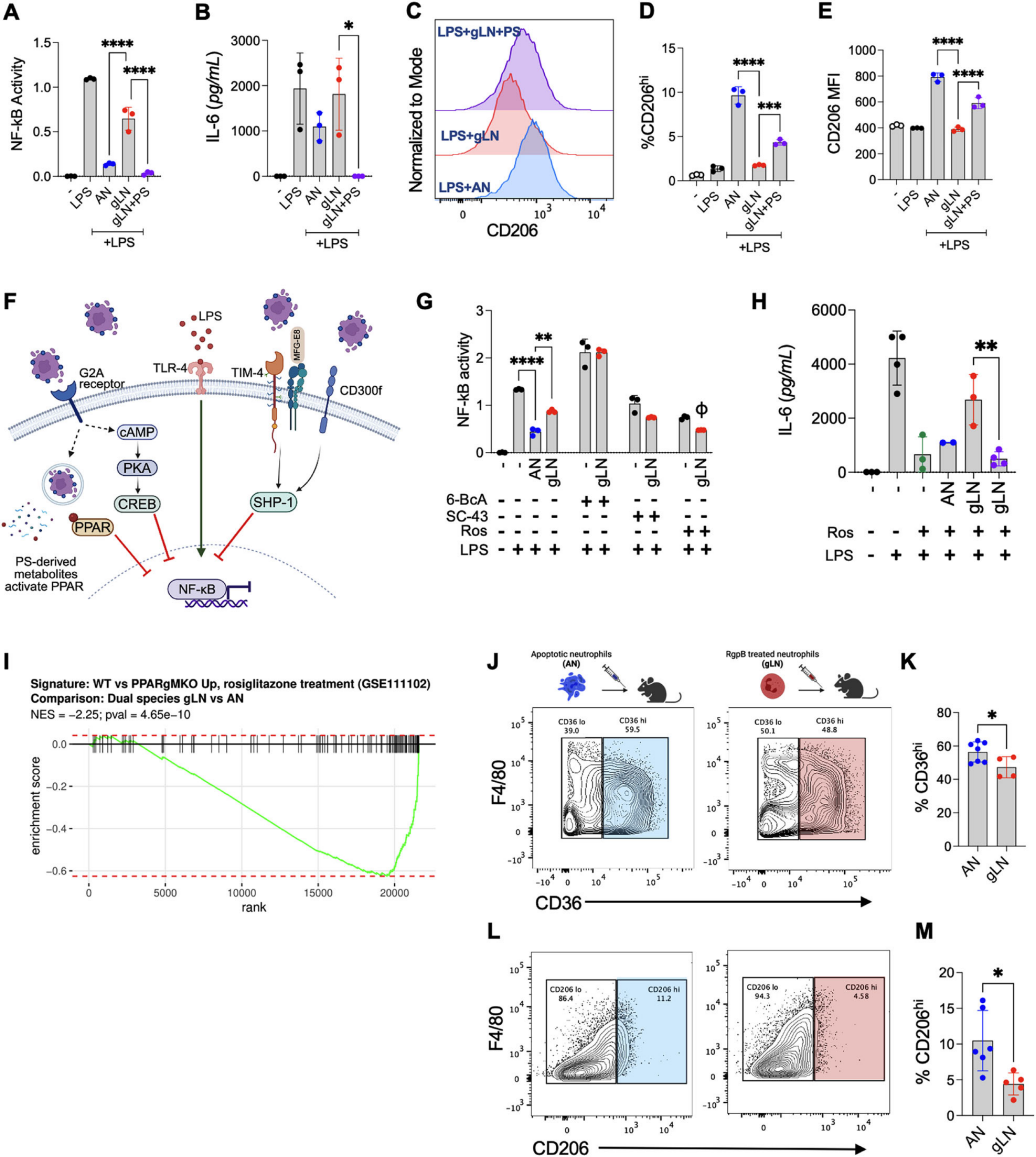
Restoring PS signaling and downstream PPAR-γ activation ameliorates inflammation perpetuated by live neutrophil entrapment. For experiments outlined in (**A**–**H**), macrophages were co-cultured with human apoptotic neutrophils (ANs), RgpB treated neutrophils (gLNs), or gLNs plus phosphatidylserine (PS) liposomes for 2 h, uningested neutrophils were removed with sequential PBS washes, and macrophages were stimulated with 10 ng/ml of LPS for 18 h. **A** NF-κB activity was measured by colorimetric quantification of secreted alkaline phosphatase (SEAP) in the NF-κB reporter RAW-Blue macrophage cell line. Data (mean ± SD) from three independent experiments are shown. **B**–**E** PEMs were pulse-fed AN or gLN ± PS liposomes followed by LPS challenge, as described above. **B** IL-6 levels in cell-free supernatants were assessed by ELISA. The surface expression of CD206 was measured by flow cytometry; **C** shows overlaid histograms while (**D**) and (**E**) show %CD206^hi^ cells and CD206 mean fluorescence intensity (MFI), respectively. Data points indicate biological replicates, and data are shown as mean ± SD. **F** Schematic depicting suppression of NF-κB by key PS receptor-mediated signaling pathways. **G** RAW-Blue macrophages were pulse-fed ANs or gLNs and stimulated with LPS (10 ng/ml) in the presence of 20 μM 6-Bnz-cAMP (6-BcA) (PKA agonist), 5 μM SC-43 (SHP-1 agonist), or 5 μM rosiglitazone (Ros) (PPAR-γ agonist) for 18 h. NF-κB activity was measured via colorimetric quantification of SEAP. Data from 3 independent experiments are shown as mean ± SD. **H** PEMs were pulse-fed AN or gLN for 2 h, stimulated with LPS ± Ros for 18 h and IL-6 levels were measured by ELISA. Data points indicate biological replicates, and data are shown as mean ± SD. Statistical significance was calculated by one-way ANOVA with Tukey’s correction: *p < 0.05, **p < 0.02, ***p < 0.001, ****p < 0.0001. **I** RNA-seq data from Fig. 4 was used to calculate the gene set enrichment plot (GSEA) for the PPAR-regulated genes in gLN- vs AN-ingesting macrophages (PEMs) in vivo. **J**–**M** 10^7^ human ANs or gLNs were injected into the inflamed peritoneum of neutropenic mice. 4 h post-injection, peritoneal cells were collected by lavage, and the protein expression of PPAR-γ regulated genes CD36 (**J**, **K**) and CD206 (**L**, **M**) in PEMS was determined by flow cytometry. Data points indicate biological replicates, and data are shown as mean ± SD. Statistical significance was calculated using an unpaired *t*-test; *p < 0.05. The illustration above, data panels in (**J**), was created with Biorender.com.

We hypothesized that the inflammatory nature of gLN entrapment was driven in part due to the lack of engagement of PS sensing receptors and PS-triggered downstream signaling that counters the activity of inflammatory transcription factors such as NF-κB [42]. Unlike ANs, gLNs, were unable to suppress LPS induced NF-κB activation in the RAW-Blue NF-κB macrophage reporter cell line (Fig. 6A). Expectedly, restoring PS signaling by adding PS liposomes abrogated NF-κB activation, downregulated the secretion of IL-6, and promoted transition towards a pro-resolving phenotype as measured by the expression of CD206 (Fig. 6A–E). These data indicated that targeting PS signaling in macrophages with entrapped neutrophils was sufficient to downregulate their inflammatory potential.

PS sensing receptors, such as TIM-4 and CD300f receptors, inhibit LPS-mediated NF-κB activation by activating Src homology protein-1 (SHP-1), a tyrosine phosphatase that prevents IκBα degradation [43–45]. Alternatively, PS or Lyso-PS sensing by G2A receptors has been shown to elevate cAMP levels, resulting in the activation of cAMP-response element-binding protein (CREB), which ultimately displaces RelA/p65 subunit, halting NF-κB mediated transcription of pro-inflammatory cytokines [46] (Fig. 6F). In addition, the catabolic breakdown of apoptotic cell-associated proteins, lipids, and fatty acids within efferosomes are degraded into metabolites that have been previously shown to activate nuclear receptors, such as peroxisome proliferator-activated receptors PPAR-γ, PPAR-δ, and liver X receptors to trans-repress NF-κB activity [47–50]. Synthetic agonists SC-43 or 6-Bnz-cAMP (6-BcA) that activate SHP-1 and PKA/CREB axis, respectively, did not block NF-κB activation in gLN efferocytosing RAW-Blue reporter macrophages; however, activation of PPAR-γ with synthetic agonist rosiglitazone (Ros) resulted in significant suppression of NF-κB activity in gLN phagocytosing macrophages (Fig. 6G). We also confirmed our findings in primary macrophages (PEMs) (Fig. 6H).

PPAR-γ activation is central to the immune suppressive nature of efferocytosis, and multiple PPAR-γ agonists have been used therapeutically to modulate inflammatory outcomes emanating from defective efferocytosis in inflammatory diseases [48, 51]. In addition to immune suppression, PPAR-γ modulates macrophage lipid homeostasis by facilitating the breakdown of fatty acids from ingested cells and boosting efferocytosis by increasing PS receptor expression [52]. Our RNA-seq data showed significantly reduced activation of the PPAR-γ signaling and lipid metabolism pathways (Fig. 6I and Fig. 4H, I) in gLN-ingesting macrophages. To determine whether loss of PPAR-γ activity correlated with reduced M2 priming of macrophages, we adoptively transferred ANs or gLN (i.p.) into the inflamed cavities of mice and determined the expression of PPAR- γ -regulated genes CD36 and CD206 [53–55] after 18 h. PEMs from gLN ingested mice show depressed expression of CD36 and CD206 (Fig. 6J–M), confirming defective activation of PPAR-γ during atypical efferocytosis in vivo. These data demonstrate that restoring PS signaling and/or PPAR-γ activity is sufficient to block the inflammatory response during the engulfment of live neutrophils.

## Discussion

An inflammatory milieu is conducive to the lifestyle of *Pg*, and it is particularly adept at surviving it. Due to its asaccharolytic nature, *Pg* relies on gingipain activity to generate amino acids as its carbon and energy source. The endopeptidase activity of gingipains effectively fragments several antimicrobial proteins such as antibodies, complement proteins, cytokines, and chemokines, liberating amino acids for nutritional needs and disarming immune effector responses [12, 56]. Neutrophils are highly abundant in the oral cavity. Thus, diminishing their effector responses by either gingipain-mediated incapacitation of neutrophil antimicrobial responses or sequestration of live neutrophils within other cell types is imperative for *Pg* survival [11]. Here, we show a novel role for gingipains in live neutrophil sequestration and, thus, removal from the milieu, which further aids *Pg* survival in vivo (Fig. S6). To our knowledge, this is the first example of live neutrophil entrapment in the context of bacterial infection.

We show that the phagocytosis of RgpB-treated live neutrophils relied on α_M_β_2_ (CD11b/CD18) integrin, a highly promiscuous heterodimeric receptor with over a hundred reported ligands [27]. While there could be several other RgpB-targeted proteins, or their cleavage products, that could potentially serve as α_M_β_2_ ligands, we focused on neutrophil granule proteins, as they have been described as interacting ligands of α_M_β_2_ integrin, and can be naturally exocytosed out of the neutrophil where they would be accessible to macrophage α_M_β_2_ receptors to facilitate uptake [27, 57]. Secondly, we focused on proteins that were enriched in neutrophils, as RgpB-treated thymocytes that lack neutrophil granule proteins but share other proteins on the TAILS list of peptides (Supplementary Tables 1, 2) did not show significant uptake (Fig. S3A–D). Thus, RgpB mediated generation of novel peptides from the degradation of neutrophil proteins is essential for live neutrophil entrapment and is unique to *Pg* pathogenesis.

Efferocytosis, or the uptake of dead cells, is critical in preventing an excessive immune response to infection [58]. Our in vivo data showed that the entrapment of live neutrophils led to significant inflammatory responses that also impacted the bystander macrophages within the tissue milieu, possibly contributing to the perpetuation of dysregulated inflammatory responses. Interestingly, unresolving low-grade inflammation is a hallmark of periodontitis and *Pg* pathogenesis and has been mechanistically linked with enhanced susceptibility to several systemic chronic inflammatory or autoimmune diseases in humans [59]. Using RNA-seq, we were able to show that macrophages with entrapped live neutrophils showed a profound failure in the activation of PPAR-γ regulated pro-resolution pathways. However, the alternate utilization of α_M_β_2_ integrin for entrapment of live neutrophils might further contribute to sustaining inflammation. Aside from being an important phagocytic/efferocytic receptor, α_M_β_2_ integrin signaling can also positively modulate TLR signaling. α_M_β_2_ integrin activation (outside-in signaling) induces phosphatidylinositol-bisphosphate (PIP2) production by phosphatidylinositol 5-kinase (PI5K), allowing for the docking of Mal adapter proteins and downstream recruitment of MyD88 for the initiation of TLR2/4 signaling and inflammatory cytokine production [60]. In the absence of PS receptor engagement, it is possible that α_M_β_2_ integrin activation might augment inflammatory outcomes during live cell efferocytosis as observed in vitro and in vivo (Figs. 3, 4). Another contributing factor could be a delay in the downstream maturation of live neutrophil containing phagosomes, resulting in delayed degradative clearance of ingested cargo. In concordance with this hypothesis, we saw significant delays in several catabolic and degradative pathway genes in macrophages with entrapped live neutrophils (Fig. 4). Incomplete degradative clearance of efferocytosed apoptotic cells has been mechanistically linked with several chronic inflammatory and autoimmune consequences, often due to the generation of self-reactive T cell clones and auto-antibody-mediated tissue destruction [42, 58]. In MCs, entrapped activated neutrophils eventually undergo apoptosis, and recycling their antimicrobial proteins increases mast cell ‘fitness’ in a model of allergic inflammation [8]. While our unpublished observations point to a slower death of entrapped neutrophils in the context of *Pg* infection, the molecular mechanisms of their clearance, metabolic and transcriptional responses, and immune consequences are beyond the scope of the current manuscript.

A limitation of our work is that we are unable to confirm our findings in the context of periodontitis due to the fastidious growth of the *ΔkgpΔrgpAΔrgpB* mutant (*ΔKRAB*) and its inability to effectively colonize the oral cavities of mice [61]. However, gingipains can circulate systemically, potentially impacting neutrophils at sites distant from the oral cavity [17, 59]. Our findings demonstrate a previously undefined role of live neutrophil entrapment during *Pg* infection as a significant contributing factor to augmenting host inflammatory burden. Furthermore, these studies provide unique insights into a previously undescribed mechanism of pathogenic manipulation of neutrophil function by *Pg*.

## Materials and methods

### Bacteria

*Porphyromonas gingivalis* (*Pg*) *ATCC* 33277, *Pg Δppad*, *Pg ΔfimA* [62], and *Pg ΔkgpΔrgpAΔrgpB* (*ΔKRAB*) [63] were cultured in brain heart infusion (BHI) broth supplemented with yeast extract (1 mg/mL), hemin (5 µg/mL), and menadione (1 µg/mL). All cultures for mutants were supplemented with appropriate antibiotics: *Δppad* and *ΔfimA* had erythromycin (5 µg/mL); *ΔKRAB* had erythromycin (5 µg/mL), tetracycline (1 µg/mL), and chloramphenicol (4 µg/mL). For specific inhibition of RgpB activity, *P. gingivalis* was incubated with 5 µM D-Phe-Phe-Arg-chloromethylketone (FFR-CMK) for 30 min at room temperature. All strains were grown anaerobically (85% N_2_, 10% H_2_, 5% CO_2_) at 37 °C.

### Human neutrophil isolation and treatments

Human peripheral blood was obtained from healthy donors, and neutrophils were purified using a discontinuous Percoll gradient, as previously described [64]. Apoptosis was induced by culturing 15 × 10^6^ cells per mL in RPMI + 5% heat-inactivated fetal bovine serum (ΔFBS) containing 50 µg/mL cycloheximide (MilliporeSigma) for 18 h. Gingipain (RgpB) treatment was done as previously described by Guzik et al. [14]. Briefly, RgpB (1.2 µM) was activated by dilution in gingipain activation buffer (20 mM HEPES, 5 mM CaCl_2_, pH 8.0; supplemented with 10 mM L-cysteine) and incubated at 37 °C for 15 min. Activated gingipains were then diluted 1:1 with RPMI to a working concentration of 600 nM. Live, freshly isolated neutrophils were suspended at 30 × 10^6^ cells per mL in RPMI+ ΔFBS and further diluted 1:1 with the working stock of activated RgpB to a final concentration of 300 nM and incubated at 37 °C, 5% CO_2_ for 1 h. After incubation, neutrophils are washed twice in RPMI to remove any residual RgpB and used for efferocytosis assays [14]. For assays requiring RgpB inhibition, activated RgpB were incubated with 5 µM D-Phe-Phe-Arg-chloromethylketone (FFR-CMK) for 5 min at room temperature prior to addition to neutrophil suspension. Inhibition was confirmed by kinetic RgpB activity assays using chromogenic substrate Nα-Benxoyl-L-arginine 4-nitroanilide hydrochloride (L-BAPNA) [65]. After cycloheximide or RgpB treatment, neutrophil viability was assessed by PS expression and necrosis by Annexin V/7-AAD staining, followed by flow cytometry [14, 15]. Live neutrophils were also challenged with *Pg* (1 neutrophil: 10 *Pg*) for 1 h. For in vivo experiments, RgpB-treated neutrophils were passed through EasySep Dead Cell Removal (Annexin V) Kit (Stem Cell Technologies) to remove any apoptotic cells.

### Mice

C57BL/6J (CD45.2) and B6.SJL-*Ptprc*^*a*^ *Pepc*^*b*^/BoyJ (CD45.1) mice were purchased from The Jackson Laboratory, and *Cybb*^-/y^ (Nox2 oxidase null) mice [66] were generated from in-house colonies. Male and female mice (between 8 and 12 weeks old) were maintained in specific pathogen-free conditions. The Institutional Animal Care and Use Committee at the University of Louisville and the Abigail Wexner Research Institute at Nationwide Children’s Hospital approved all animal experiments.

### Efferocytosis assays

Apoptotic or RgpB-treated neutrophils of human or mouse origin were suspended in RPMI and pulse-fed to PEMs at a ratio of 10 neutrophils: 1 macrophage for 2 h. Uningested neutrophils were removed by three sequential washes with sterile PBS. For experiments with liposomes, 5 µmol PS liposomes (1-oleoyl-2-hydroxy-sn-glycero-3-phospho-L-serine; Avanti Polar Lipids) were added in addition to gLNs. In certain experiments, relative efferocytic rates were determined by neutrophil MPO staining of PEMs with 3,3′-diaminobenzidine (DAB). After staining, at least 300 total macrophages were counted (blinded) per condition, and % MPO positivity was determined [15]. For flow cytometry-based efferocytosis assays, neutrophils were labeled either with PKH26-PCL (MilliporeSigma) or CellTrace Violet (ThermoFisher) as per the manufacturer’s instructions. After pulse feeding, PEMs were identified with the macrophage specific marker F4/80 (Clone BM8; BioLegend) and anti-human CD45 (Clone HI30; BioLegend), or anti-mouse CD45.1 (Clone A20; BioLegend), or anti-Ly6G (Clone 1A8; BD Biosciences) to exclude extracellular neutrophils. For in vivo estimation of efferocytosis, relative uptake was determined in F4/80^+^ PEMs 4 h post gLN or AN injection by measuring CellTrace Violet positivity (ThermoFisher) or intracellular staining with anti-human MPO antibody (Clone 2C7; Abcam). Total lavage cells were stained and analyzed by collecting an equal number of events on LSR Fortessa (BD Biosciences) and gating on F4/80^+^, Ly6G^-^, CellTrace Violet^+,^ or F4/80^+^, hCD45^-^, MPO^+^ cells.

### Peritonitis and peritoneal exudate macrophage (PEM) isolation

Mice were injected intraperitoneally (i.p.) with 1 mL of sterile 5 mM sodium metaperiodate (MilliporeSigma) solution in saline to induce peritonitis [15, 67]. For all in vivo experiments, gLNs or ANs were adoptively transferred (i.p) after 72 h. For in vitro experiments, mice were euthanized at 72 h, and the peritoneal cavities were serially lavaged with sterile, cold PBS + 2 mM EDTA. PEMs were adherence purified by plating total lavage cells in tissue culture plates in DMEM containing 10% ΔFBS at 37 °C, 5% CO_2_ for 2 h, followed by the removal of non-adherent cells. For all efferocytosis assays, PEMs were rested overnight before pulse feeding with neutrophils.

A complete list of methods is in the supplemental materials.

## Supplementary information

Supplementary Materials

Supplemental Figure S1

Supplemental Figure S2

Supplemental Figure S3

Supplemental Figure S4

Supplemental Figure S5

Supplemental Figure S6

Supplemental Tables

## Data Availability

All data generated or analyzed during this study are included in this published article (and its supplementary information files). The mass spectrometry proteomics data have been deposited to the ProteomeXchange Consortium via the PRIDE partner repository with the dataset identifier PXD051073 [68]. All data about identified and quantified peptides and proteins are included in Supplementary Tables 1 and 1. RNA-seq datasets are deposited in the NCBI GEO database (GSEA264524).
