# Supplementary material for: The trapping of live neutrophils by macrophages during infection
Source: Cell Death Dis. 2025 Jul 3;16(1):488. doi: 10.1038/s41419-025-07808-5 (PMC12229712; doi:10.1038/s41419-025-07808-5)
Supplement: Supplementary file 1 — Supplementary Materials [file 41419_2025_7808_MOESM1_ESM.docx]

**SUPPLEMENTAL METHODS**

**In vivo neutrophil depletion.** Endogenous neutrophil populations were depleted in C57BL/6J mice by i.p. injection of anti-Ly6G (1A8) (100 μg/mL) and anti-rat kappa light chain (100 μg/mL) as described ^1, 2^ prior to the induction of peritonitis (described above). Neutrophil depletion was confirmed in peripheral blood (submandibular bleed) using Gr-1 (Clone RB6-8C5; BioLegend) and CD11b (Clone M1/70; BD Biosciences) antibodies and flow cytometry.

**Murine bone marrow neutrophil isolation and treatments:** Total bone marrow was extracted by flushing femurs and tibia with serum-free RPMI and bone marrow neutrophils (BMNs) isolated using the EasySep Mouse Neutrophil Enrichment Kit (Stem Cell Technologies) as per manufacturer’s protocols. Apoptosis induction and RgpB treatment was done as described in the human neutrophil isolation section above.

**Thymocyte isolation and treatments.** Thymus glands were collected from 5-week-old C57BL/6J mice and thymocytes isolated by digestion in sterile HBSS (Gibco) supplemented with 5 mM Collagenase IV (Gibco) in the Cytiva VIA Extractor Tissue Disaggregator (240 rpm, 15 minutes, 37⁰C). Thymocytes were labeled with PKH26 (MilliporeSigma) as per manufacturer’s instructions. Thymocytes were exposed to UV radiation for 5 minutes to induce apoptosis, then cultured for 2 hours in RPMI + 5% ΔFBS at 37⁰C, 5% CO_2_. Separately, thymocytes, similar to neutrophils, were treated with RgpB as described in the “Human neutrophil isolation and treatments” section. Prior to pulse feeding to macrophages, untreated and RgpB -treated thymocytes were passed through EasySep Dead Cell Removal (Annexin V) Kit (Stem Cell Technologies), to remove apoptotic cells as per the manufacturer’s instructions.

**Cytokine responses:** PEMs were pulse-fed ANs or gLNs for 2 h, then incubated with LPS (10 ng/mL) for 18 h. Cytokine levels in cell-free supernatants were profiled by Milliplex Mouse Cytokine/Chemokine multiplex assay (MilliporeSigma) in the University of Louisville Microbiomics Core using Luminex 100 machine. For statistical analysis of groups that exceed the limit of detection, the upper value was assigned. For specific experiments, IL-6 levels were determined by ELISA (R&D Systems) according to the manufacturer’s instructions and measured using a BMG LabTech microplate reader.

**RAW-Blue macrophage culture.** RAW-Blue macrophages (NF-κB SEAP reporter cell line) were obtained from Invivogen and maintained in culture in DMEM + 10% ΔFBS supplemented with 100 µg/ml normocin and 100 µg/ml zeomycin (Invivogen). NF-κB activity was determined in cell-free supernatants using Quanti-Blue substrate, at λ_620nM_ using a BMG LabTech microplate reader.

**Confocal microscopy and MPO fluorescent staining:** Human neutrophils (before and after RgpB treatment) were incubated on poly-L-lysine (MilliporeSigma) coated glass slides for 2 h, then fixed with 2% paraformaldehyde. Cells were *not* permeabilized prior to staining. Wheat Germ Agglutinin-AlexaFluor 647 (ThermoFisher) was used for membrane labeling, according to the manufacturer’s protocol. Then, cells were blocked with 5% BSA (1 h; room temperature) and stained with unconjugated primary rabbit anti-human MPO antibody (Clone E1E7I; Cell Signaling Technology) for 18 h at 4⁰C. Slides were washed three times with PBS prior to secondary antibody staining. Secondary antibody staining used goat anti-rabbit IgG (H+L) cross-adsorbed secondary antibodies (AlexaFluor 488 or AlexaFluor 647; Invitrogen) for 2 h at room temperature and 4’,6-diamidino-2-phenylindole (DAPI; MilliporeSigma) for nuclear staining. Slides were mounted with ProLong Gold Antifade Mountant (Invitrogen). Slides were imaged using a Nikon AX R confocal microscope and analyzed with NIS-Elements Software (Nikon).

**Flow Cytometry.** For all experiments, prior to the addition of flow antibodies, Fc receptors were blocked using 2.4G2 supernatants or 0.1% pooled human AB serum for mouse and human cells, respectively (10 min at RT). Macrophage polarization was determined by staining PEMs with fluorescently conjugated antibodies against CD80 (Clone 16-10A1; BioLegend), CD36 (Clone CRF D-2712; BD Biosciences), CD206 (Clone C068C2; BioLegend), and F4/80 (Clone BM8; BioLegend) for 45 minutes at 4°C in flow buffer (PBS + 2 mM EDTA + 1% BSA), followed by analysis with FACS Celesta (BD Biosciences). Neutrophil apoptosis was determined by staining with Annexin V (BioLegend) for 30 minutes at 4⁰C in 1x Annexin V Binding Buffer (BD Biosciences). Neutrophil surface CD63, MPO, and elastase expression was determined by staining with anti-human CD63 (Clone H5C6; Biolegend), anti-human MPO (Clone 2C7; Abcam), and anti-human neutrophil elastase (Clone E9C9L; Cell Signaling Technology) for 45 minutes at 4°C in flow buffer. Data were acquired on BD Celesta or Fortessa and analyzed using Flowjo Software. A detailed list of all antibodies used for flow staining is provided in the reagent table below.

**Ligature induced periodontitis**: A 5.0 silk ligature was placed on the second maxillary molar of C57BL/6J mice as previously described ^3^. On alternate days following ligature placement, mice were intravenously (i.v.) injected (tail vein) with 10^7^ RgpB-treated murine bone marrow neutrophils or sterile saline (control). After 7 days, mice were euthanized, and heads extracted and preserved in 10% formalin before scanning using microcomputed tomography (µCT). Average bone loss was measured by taking linear measurements (in millimeters) from the cemento-enamel junction (CEJ) to the alveolar bone crest (ABC) in the interdental regions between the first and second molars (M1-M2) or the second and third molars (M2-M2’), as previously described by Park et al. ^4^ using ImageJ.

***In vivo* bacterial survival**: Sterile peritonitis was experimentally induced in C57BL/6J mice by intraperitoneal (i.p.) injection of 1 mL of sterile 5 mM sodium metaperiodate (Millipore Sigma) ^5^. 72 h later, 10^7^ live untreated neutrophils (LNs) or RgpB-treated live neutrophils (gLNs) were adoptively transferred (i.p) to allow for uptake by phagocytes. After 2 h, 10^8^ *P. gingivalis* (WT) was directly injected i.p. All mice were euthanized 2 h after bacterial injection, peritoneal cavities were lavaged with sterile PBS with 2 mM EDTA, and peritoneal lavage fluid was plated on blood agar plates to estimate surviving bacteria. After 5-7 days of anaerobic culture, CFUs were enumerated.

**Dual species RNA seq.** Endogenous neutrophils were depleted by i.p. injection of anti-Ly6G (1A8) (100 μg/mL) and anti-rat kappa light chain (100 μg /mL) as described in ^1, 2^ and peritonitis was induced by i.p. injection of 1 mL of sterile 5 mM sodium metaperiodate (Millipore Sigma) ^5^. After 72 h, mice were injected i.p. with 10^7^ human gLN or AN, euthanized at 4 h, and peritoneal cells lavaged using sterile PBS with 2 mM EDTA. Cells were stained with fluorescently conjugated anti-mouse antibodies against TCRβ (Clone H57-597; BD Biosciences), CD19 (Clone 1D3; BD Biosciences), Siglec F (Clone E50-2440; BD Biosciences), Ly6G (Clone 1A8; BioLegend), CD115 (Clone AFS98; BD Biosciences), F4/80 (Clone BM8; BioLegend). After lineage depletion gate (TCRβ^-^, CD19^-^, Siglec F^-^, and Ly6G^-^) cells were sorted by BD Influx Cell Sorter (BD Biosciences) to obtain a pure macrophage (F4/80^int-hi^, CD115^+^) population as shown in **Figure S5A**. Immediately after sorting, RNA was collected using RNeasy Mini Kit (Qiagen) and sent to Novogene Corporation (Sacramento, CA) for RNA-sequencing. Pair-end reads coming from bulk RNA-seq of single-species experiments were aligned with STAR ^6^ v2.7.0f. Gene counts were derived from the number of uniquely aligned unambiguous reads by featureCount ^7^ v2.0.0. Alignment and gene counts were generated against the GRCm39 (GENCODE release 33) genome assembly. RNA alignment metrics were assessed with CollectRnaSeqMetrics function from Picard ^8^ v2.21.1. The DESeq2 computational pipeline ^9^ v1.30.1 was used to normalize counts and perform differential expression analysis. The model design included condition and replicate information. Genes with less than 10 counts were filtered out. PCA plots were created using plotPCA function from DESeq2 ^9^ v1.30.1 after variance stabilizing transformation. For PCA and further analysis, only 4-hour samples were used. Gene set enrichment analysis was performed on the pre-ranked list based on Wald statistic for DESeq2 ^9^ outputs using the R package fgsea ^10^ v1.25.2. Pathway enrichment was performed using the canonical pathway database collection accessed through msigdb R package ^11^ v7.2.1. Enrichment plots were created using plotEnrichment function from fgsea ^10^ v1.25.2 package. Bar plots were created using ggplot2 package ^12^ v3.3.2. Heatmaps were created using the phantasus ^13^ web-application. Statistical significance for differentially expressed (DE) genes and pathways can be found in Tables 1-4.

**Single species RNA-seq.** Peritonitis was induced in CD45.2 recipient mice as described above. After 48 h, mice were injected intraperitoneally with PKH-PCL (MilliporeSigma) to in vivo label recruited PEMs and other phagocytes. Next day, BMNs were isolated from CD45.1 donor mice, labeled with CellTrace Violet (ThermoFisher), then incubated with RgpB for 1 h (g-BMN) or 5 μg/ml cycloheximide for 18 h to induce apoptosis (a-BMN). 10^7^ g-BMN or a-BMN were injected i.p. 72 h after induction of peritonitis. After 4 h, mice were euthanized, and peritoneal leukocytes were collected by lavage. Cells were stained with TCRβ (Clone H57-597; BD Biosciences), CD19 (Clone 1D3; BD Biosciences), Siglec F (Clone E50-2440; BD Biosciences), Ly6G (Clone 1A8; BioLegend), CD115 (Clone AFS98; BD Biosciences), F4/80 (Clone BM8; BioLegend), CD45.1 (Clone A20; BD Biosciences) and subjected to two-way sort in BD Influx Cell Sorter (BD Biosciences). After lineage depletion gate (TCRβ^-^, CD19^-^, Siglec F^-^, and Ly6G^-^), macrophages (PKH^+^, CD115^+^, F4/80^+^) were sorted based on CellTrace Violet positivity into two populations of ‘control’ or non-efferocytosing macrophages (CellTrace Violet negative) and efferocytosing macrophages (CellTrace Violet positive macrophages) as shown in **Figure S5B**. Un-ingested neutrophils were excluded by CD45.1^-^ gating. We also sorted PEMs from uninjected control mice. Immediately after sorting, RNA was isolated using RNeasy Micro Kit (Qiagen). Ultra-low input RNA sequencing was done at the GTAC core at Washington University in St. Louis. Pair-end reads from bulk RNA-seq of single-species experiments were aligned with STAR ^6^ v2.7.9a1. Gene counts were derived from the number of uniquely aligned unambiguous reads by featureCount ^7^ v2.0.32. Alignment and gene counts were generated against the GRCm38.p6 (Ensembl release 101) mouse genome assembly. Sequencing performance was assessed for the total number of aligned reads, total number of uniquely aligned reads, and features detected. The ribosomal fraction, known junction saturation, and read distribution over known gene models were quantified with RSeQC ^14^ v4.04. Differential expression, pathways analysis, and visualization were performed using methods described in the dual species part of the analysis. The model design for DESeq2 ^9^ v1.30.1 included condition information. PCA plot was built without one sample which was considered as an outlier (pem condition, replicate 3). Statistical significance for differentially expressed (DE) genes and pathways can be found in Tables 5-10.

**N-terminomics/terminal isotopic labeling (TAILS) ^15^:**

Freshly isolated live neutrophils from 3 independent donors were untreated (Buffer) or treated with RgpB (gLNs) as described above. LN and gLN cell pellets were treated with guanidine HCl (6 M; pH 8.0) prior to N-terminomics. Samples were reduced with 5 mM dithiothreitol (DTT) for 1 h at 37° C and alkylated with 15 mM iodoacetamide (IAA) for 30 minutes at room temperature, and then quenched with DTT (15 mM). pH was adjusted to 6.5, then RgpB-treated samples were isotopically labeled with deuterated heavy formaldehyde (40 mM) and untreated (Buffer) controls were treated with light formaldehyde (40 mM) overnight at 37⁰C, then precipitated using acetone-methanol (8:1). The precipitated pellet was resuspended in 1 M NaOH. Proteins were digested with trypsin overnight at 37 °C. pH of all samples was adjusted to 6.5, then incubated overnight at 37 °C with dendritic polyglycerol aldehyde polymer. Unbound peptides were removed by passing through a centrifugal filter unit (10 kDa limit in membrane) with centrifugation at 10,000 x *g*, 5 minutes. The membrane was serially washed 3 times with Tris-HCl (100 mM, pH 6.5). Samples were adjusted to a pH of 3 with 100% formic acid. All samples were desalted with Sep-Pak C18 columns, then lyophilized for analysis by liquid chromatography-mass spectrometry (LC-MS/MS; Mass Spectrometry Core Facility, University of Calgary, Canada).

## **High performance liquid chromatography (HPLC) and mass spectrometry**

All liquid chromatography and mass spectrometry experiments were carried out by the Southern Alberta Mass Spectrometry core facility at the University of Calgary, Canada. Analysis was performed on an Orbitrap Fusion Lumos Tribrid mass spectrometer (Thermo Fisher Scientific, Mississauga, ON) operated with Xcalibur (version 4.0.21.10) and coupled to a Thermo Scientific Easy-nLC (nanoflow Liquid Chromatography) 1,200 system. Tryptic peptides (2 µg) were loaded onto a C18 trap (75 µm$\times$ 2 cm; Acclaim PepMap 100, P/N 164946; Thermo Fisher Scientific) at a flow rate of 2 µL/min of solvent A (0.1% formic acid and 3% acetonitrile in LC-mass spectrometry grade water). Peptides were eluted using a 120 min gradient from 5 to 40% (5% to 28% in 105 min followed by an increase to 40% B in 15 min) of solvent B (0.1% formic acid in 80% LC-mass spectrometry grade acetonitrile) at a flow rate of 0.3% µL/min and separated on a C18 analytical column (75 µm $\times$ 50 cm; PepMap RSLC C18; P/N ES803; Thermo Fisher Scientific). Peptides were then eletrosprayed using 2.3 kV into the ion transfer tube (300° C) of the Orbitrap Lumos operating in positive mode. The Orbitrap first performed a full mass spectrometry scan at a resolution of 120,000 FWHM to detect the precursor ion having a mass-to-charge ratio (m/z) between 375 and 1,575 and a +2 to +4 charge. The Orbitrap AGC (Auto Gain Control) and the maximum injection time were set at 4 x 10^5^ and 50 ms, respectively. The Orbitrap was operated using the top speed mode with a 3s cycle time for precursor selection. The most intense precursor ions presenting a peptidic isotopic profile and having an intensity threshold of at least 2 x 10^4^ were isolated using the quadrupole (isolation window of m/z 0.7) and fragmented with HCD (38% collision energy) in the ion routing Multipole. The fragment ions (MS2) were analyzed in the Orbitrap at a resolution of 15,000. The AGC, the maximum injection time, and the first mass were set at 1 10^^5^, 105 ms, and 100 ms, respectively. Dynamic exclusion was enabled for 45 s to avoid the acquisition of the same precursor ion having a similar m/z (±10 ppm).

## **Proteomic data and bioinformatic analysis**

Spectral data were matched to peptide sequences in the human UniProt protein database using the MaxQuant software package v.1.6.0.1, peptide-spectrum match false discovery rate (FDR) of < 0.01 for the shotgun proteomics data and < 0.05 for the N-terminomics/TAILS data. Search parameters included a mass tolerance of 20 p.p.m. for the parent ion, 0.05 Da for the fragment ion, carbamidomethylation of cysteine residues (+57.021464), variable N-terminal modification by acetylation (+42.010565Da), and variable methionine oxidation (+15.994915Da). For the shotgun proteomics data, cleavage site specificity was set to Trypsin/P (search for free N-terminus and only for lysines), with up to two missed cleavages allowed. For the N-terminomics/TAILS data, the cleavage site specificity was set to semi-ArgC (search for free N-terminus) for the TAILS data and was set to ArgC for the preTAILS data, with up to two missed cleavages allowed. Significant outlier cut-off values were determined after log(2) transformation by boxplot-and-whiskers analysis using the BoxPlotR tool. Database searches were limited to a maximal length of 40 residues per peptide. Peptide sequences matching reverse or contaminant entries were removed.

**Reactome Pathway Analysis**

To identify interconnectivity among proteins, the STRING-db (Search Tool for the Retrieval of Interacting Genes) database was used to identify interconnectivity among proteins. The protein-protein interactions are encoded into networks in the STRING.v11 database (<https://string-db.org>). Metascape ([https://metascape.org](https://metascape.org/)) analysis was used to identify changes in functional enrichment, interactome analysis, and gene annotation. Our data were analyzed using *Homo sapiens* as our model organism at a false discovery rate of 1%.

**Heatmaps of cleavage sites, TopFIND and TopFINDer analysis.**

WebPICS was used using the website <http://clipserve.clip.ubc.ca/pics>. TopFIND ^16^and TopFINDer analyses were performed using the Web site http://clipserve.clip.ubc.ca/topfind/. Uniprot (<https://www.uniprot.org/>) and MEROPS (<https://www.ebi.ac.uk/merops/index.shtml>) were used to interpret the data.

**
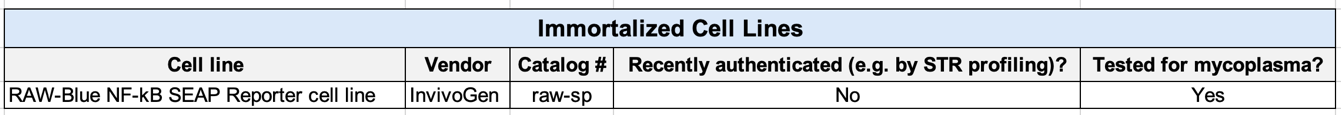

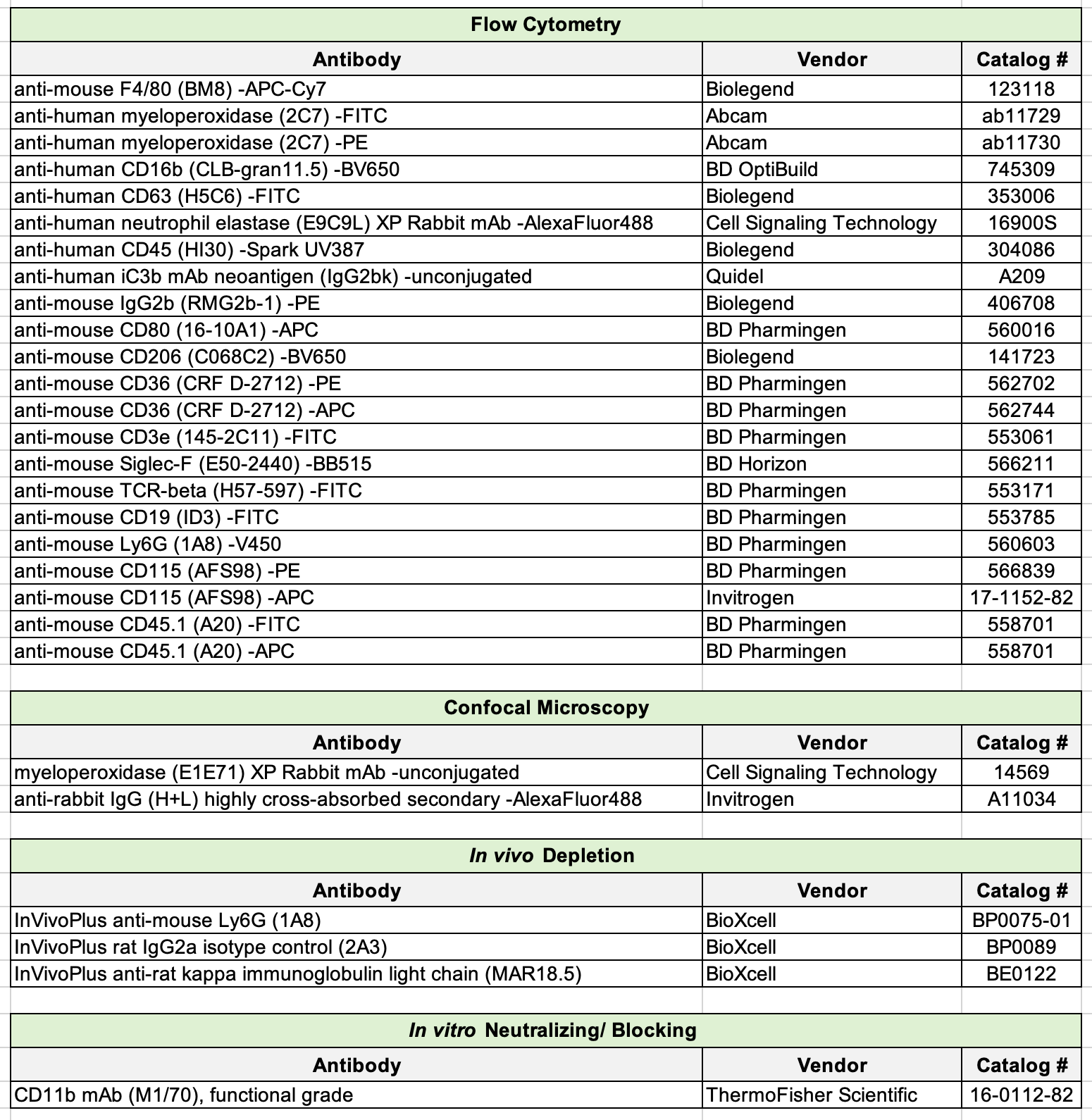
Reagent Table:**

**SUPPLEMENTAL FIGURE LEGENDS**

**Supplemental Fig. S1: RgpB is an essential virulence factor for *P. gingivalis* mediated efferocytosis of live neutrophils**

Live human neutrophils (LNs) were infected with either wildtype (WT) *Pg, Δppad, ΔfimA,* or *ΔrpgaΔrgpbΔkgp triple mutant* (*ΔKRAB*) for 1 h at an MOI of 10. Apoptotic human neutrophils (ANs) were generated as described in the methods. (**A**) Representative histograms showing estimation of phosphatidylserine (PS) externalization as assessed by flow cytometry. The phagocytosis of infected neutrophils by murine peritoneal exudate macrophages (PEMs) after 2 h of co-culture was determined by myeloperoxidase (MPO) staining as indicated by red arrows (**B**), and % uptake was quantified by scoring ~300 PEMs per group (**C**). Human neutrophils were treated for 1 h with purified active RgpB (gLN); Phe-Phe-Arg-chloromethylketone (FFR-CMK) inhibited RgpB, Pg, or FFR-CMK inhibited *Pg*. (**D**) PS expression and (**E**) % uptake was determined. Data (mean ± SD) from 2-3 experiments are shown, and statistical significance was determined using one-way ANOVA and Tukey’s correction: **p<0.02, ****p<0.0001.

**Supplemental Fig. S2: Live neutrophil entrapment is independent of species differences**

(**A**) PEMs were co-cultured with murine apoptotic or RgpB-treated bone marrow neutrophils (a-BMN or g-BMN, respectively) for 2 h, and % uptake was estimated by MPO staining and quantification (described in S1). Combined data from 3 experiments are shown as mean ± SD, and each data point represents a biological replicate. (**B**) Peritonitis was induced in WT mice (Day 0), then phagocytes were labeled with an injection of PKH-PCL (Day 2). 24 h later (Day 3), 10^7^ CellTrace Violet-labeled a-BMN or g-BMN were injected i.p. directly into the peritoneal cavity. After 4 h, the uptake rate was measured by flow cytometry by gating on Ly6G^-^, F4/80^+^, PKH^+^, and CellTrace Violet^+^ populations. Data point indicates biological replicates. Data from 3-4 mice are shown as mean ± SD; statistical significance was calculated by one-way ANOVA and Tukey’s correction. The illustration above, data panels in (B), was created with Biorender.com.

**Supplemental Fig. S3: RgpB-mediated live cell entrapment is neutrophil specific**

(**A, B**) Phosphatidylserine (PS) expression was assessed by Annexin V staining in murine live untreated thymocytes (L-thym; blue histogram), RgpB-treated live thymocytes (gL-thym; red histogram), or apoptotic thymocytes (A-thym; green histogram). (**C, D**) PKH26-labeled L-thym, gL-thym, or A-thym co-cultured PEMs for 2 h, and phagocytosing macrophages were identified by flow cytometry. Anti-mouse TCR-β and CD3e were used to exclude uningested or attached thymocytes. Ingesting macrophages were identified by gating on F4/80^int-hi^, PKH26^+^, and TCR-β^-^ cells. Data points refer to biological replicates. Data is depicted as mean ± SD, and statistical significance was calculated using a one-way ANOVA and Tukey’s correction; **p<0.01. (**E, F**) iC3b expression in apoptotic and RgpB-treated human neutrophils as measured by flow cytometry. Data points indicate biological replicates. Data are shown as mean ± SD; statistical significance was calculated using an unpaired t-test; *p<0.05. (**G**) RgpB activity assay on cell-free supernatants from neutrophils treated with inactive RgpB or active RgpB in the presence and absence of small molecule inhibitor nexinihib20 (nex). Data are representative of 3 independent experiments.

**Supplemental Fig. S4: Macrophage inflammatory priming in response to ingestion of gLNs, ANs, and *P. gingivalis*-infected neutrophils**

(**A**) PEMs were pulse-fed gLNs or ANs for 2 h, uningested cells were removed, and then incubated with LPS (10 ng/mL) for 18 h. Cell-free supernatants were analyzed for inflammatory mediators by a 32-plex cytokine array. Select cytokine and chemokine panels are shown. The dotted line indicates the level of detection, and each data point indicates a biological replicate. (**B-D**) PEMs were co-cultured with *Pg*-infected neutrophils (MOI 1:10) for 2h, followed by overnight LPS stimulation. (**B**) IL-6 levels in cell-free supernatants was determined by ELISA and (**C-D**) CD206 expression was determined by flow cytometry. Each data point indicates an individual biological replicate. Data are shown as mean ± SD. Statistical significance was calculated using one-way ANOVA and Tukey’s correction; *p<0.05, ****p<0.0001.

**Supplemental Fig. S5: *In vivo* sorting strategies**

(**A**) *In vivo* dual-species sorting strategy: 10^7^ human ANs or gLNs were injected into the inflamed peritoneum of Ly6G-depleted WT mice. 4 h after injection, mice were lavaged and peritoneal cells were sorted to obtain a pure macrophage population. After singlet gating, Siglec-F^+^, TCRβ^+^, CD19^+^, Ly6G^+^ cells were excluded. Macrophages were sorted based on F480^+^ CD115^+^ positivity. PEMs staining positive for human CD45 (hCD45^+^) were gated out to exclude any externally associated human neutrophil-macrophage doublets. (**B**) *In vivo* single-species sorting strategy: 10^7^ murine (CD45.1^+^, CellTrace Violet^+^) apoptotic or RgpB-treated bone marrow neutrophils were injected into the inflamed peritoneum of CD45.2^+^ recipient mice. After 4 h, peritoneal cavities were lavaged, and peritoneal cells were sorted to obtain two distinct pure macrophage populations. After singlet gating, Siglec-F^+^, TCRβ^+^, CD19^+^, Ly6G^+^ cells were excluded. Macrophages (F480^+^ CD115^+^) were sorted into non-ingesting ‘bystander’ or ingesting macrophages based on CellTrace Violet positivity. Population #1, non-ingesting ‘bystander macs’: F480^+^ CD115^+^, PKH26^+^, CellTrace Violet^-^. Population #2, ‘ingesting macs’: Siglec-F^-^, TCRβ^+^, CD19^-^, Ly6G^-^, F480^+^ CD115^+^, PKH26^+^, CellTrace Violet^+^, CD45.1^-^.

**Supplemental Fig. S6: Entrapment of live neutrophils by macrophages increases bacterial survival *in vivo***

(**A**) Schematic depicting experimental design was created using Biorender.com. 10^7^ untreated live neutrophils (LN) or RgpB treated live neutrophils (gLN) were injected into the inflamed peritoneum of WT mice. 2 h later, *Pg* (10^8^ CFU) were injected i.p. After 2 h, the peritoneal lavage fluid was plated, and *Pg* survival was determined by quantifying CFUs (**B**). A data point refers to a biological replicate. Data (mean ± SD) shown from 2 independent experiments. Statistical significance was calculated using an unpaired t-test; *p<0.05.

**SUPPLEMENTAL REFERENCES**

1. Boivin G, Faget J, Ancey PB, Gkasti A, Mussard J, Engblom C*, et al.* Durable and controlled depletion of neutrophils in mice. *Nat Commun* 2020, **11**(1)**:** 2762.

2. Song Z, Bhattacharya S, Huang G, Greenberg ZJ, Yang W, Bagaitkar J*, et al.* NADPH oxidase 2 limits amplification of IL-1beta-G-CSF axis and an immature neutrophil subset in murine lung inflammation. *Blood Adv* 2023, **7**(7)**:** 1225-1240.

3. Abe T, Hajishengallis G. Optimization of the ligature-induced periodontitis model in mice. *J Immunol Methods* 2013, **394**(1-2)**:** 49-54.

4. Park CH, Abramson ZR, Taba M, Jr., Jin Q, Chang J, Kreider JM*, et al.* Three-dimensional micro-computed tomographic imaging of alveolar bone in experimental bone loss or repair. *J Periodontol* 2007, **78**(2)**:** 273-281.

5. Bagaitkar J, Pech NK, Ivanov S, Austin A, Zeng MY, Pallat S*, et al.* NADPH oxidase controls neutrophilic response to sterile inflammation in mice by regulating the IL-1alpha/G-CSF axis. *Blood* 2015, **126**(25)**:** 2724-2733.

6. Dobin A, Davis CA, Schlesinger F, Drenkow J, Zaleski C, Jha S*, et al.* STAR: ultrafast universal RNA-seq aligner. *Bioinformatics* 2013, **29**(1)**:** 15-21.

7. Liao Y, Smyth GK, Shi W. featureCounts: an efficient general purpose program for assigning sequence reads to genomic features. *Bioinformatics* 2014, **30**(7)**:** 923-930.

8. Picard Toolkit. 2019 [cited]Available from: <https://broadinstitute.github.io/picard/>

9. Love MI, Huber W, Anders S. Moderated estimation of fold change and dispersion for RNA-seq data with DESeq2. *Genome Biol* 2014, **15**(12)**:** 550.

10. Korotkevich G SV, Budin N, Shpak B, Artyomov MN, Sergushichev A. Fast gene set enrichment analysis

. *bioRxiv* 2019.

11. Bhuva D SG, Garnham A. msigdb: An ExperimentHub Package for the Molecular Signatures Database (MSigDB). R package.; 2023.

12. H W. *ggplot2: Elegant Graphics for Data Analysis*. Springer-Verlag New York, 2016.

13. Maksim Kleverov DZ, Vladislav Kamenev, Margarita Sablina, Maxim N Artyomov, Alexey A Sergushichev Phantasus, a web-application for visual and interactive gene expression analysis. *eLife* 2024, **13:e85722**.

14. Wang L, Wang S, Li W. RSeQC: quality control of RNA-seq experiments. *Bioinformatics* 2012, **28**(16)**:** 2184-2185.

15. Das N, de Almeida LGN, Derakhshani A, Young D, Mehdinejadiani K, Salo P*, et al.* Tryptase beta regulation of joint lubrication and inflammation via proteoglycan-4 in osteoarthritis. *Nat Commun* 2023, **14**(1)**:** 1910.

16. Fortelny N, Yang S, Pavlidis P, Lange PF, Overall CM. Proteome TopFIND 3.0 with TopFINDer and PathFINDer: database and analysis tools for the association of protein termini to pre- and post-translational events. *Nucleic Acids Res* 2015, **43**(Database issue)**:** D290-297.
