# Supplementary figures and images for: The trapping of live neutrophils by macrophages during infection
Source: Cell Death Dis. 2025 Jul 3;16(1):488. doi: 10.1038/s41419-025-07808-5 (PMC12229712; doi:10.1038/s41419-025-07808-5)

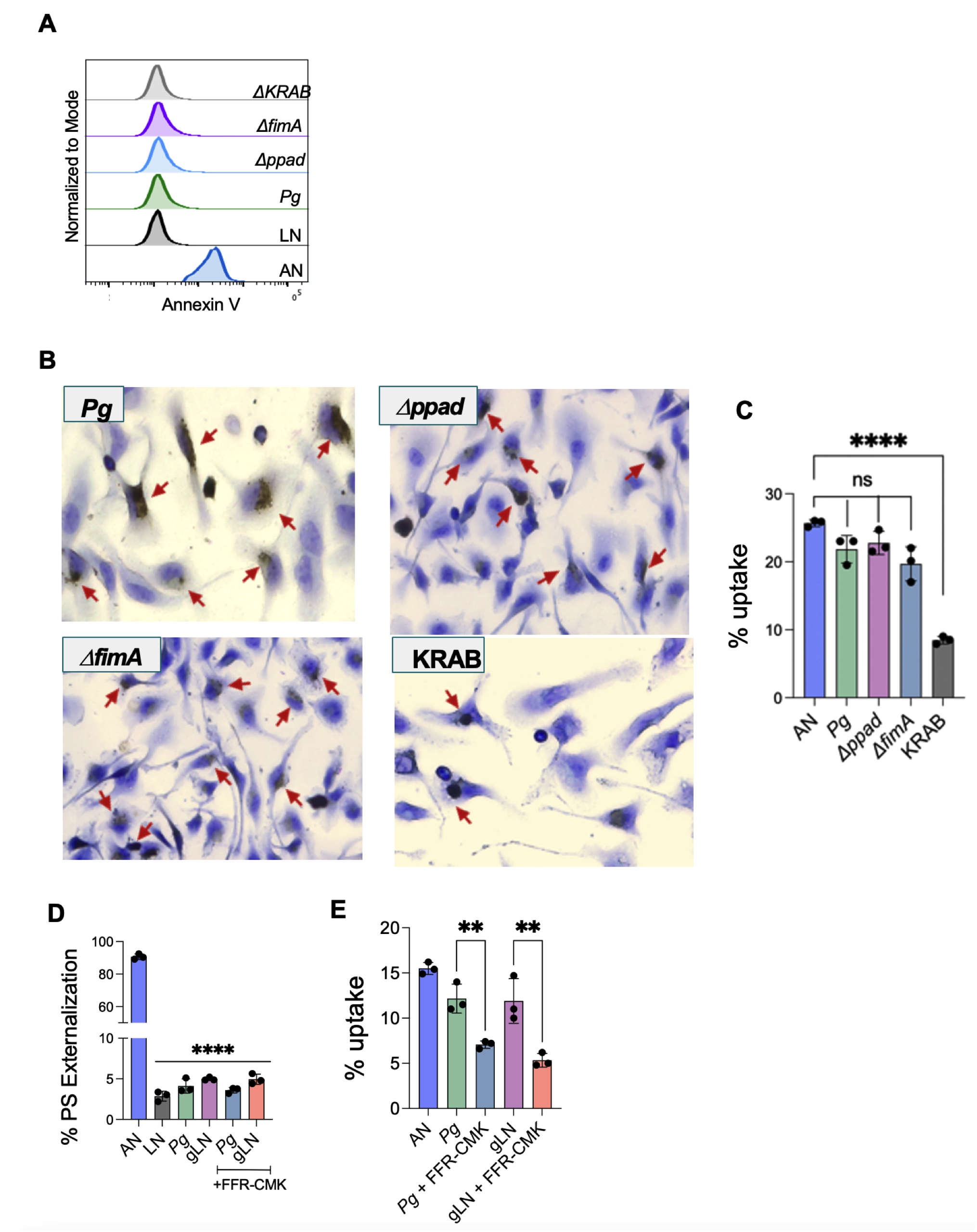

Supplement: Supplementary file 2 — Supplemental Figure S1 [file 41419_2025_7808_MOESM2_ESM.tif]

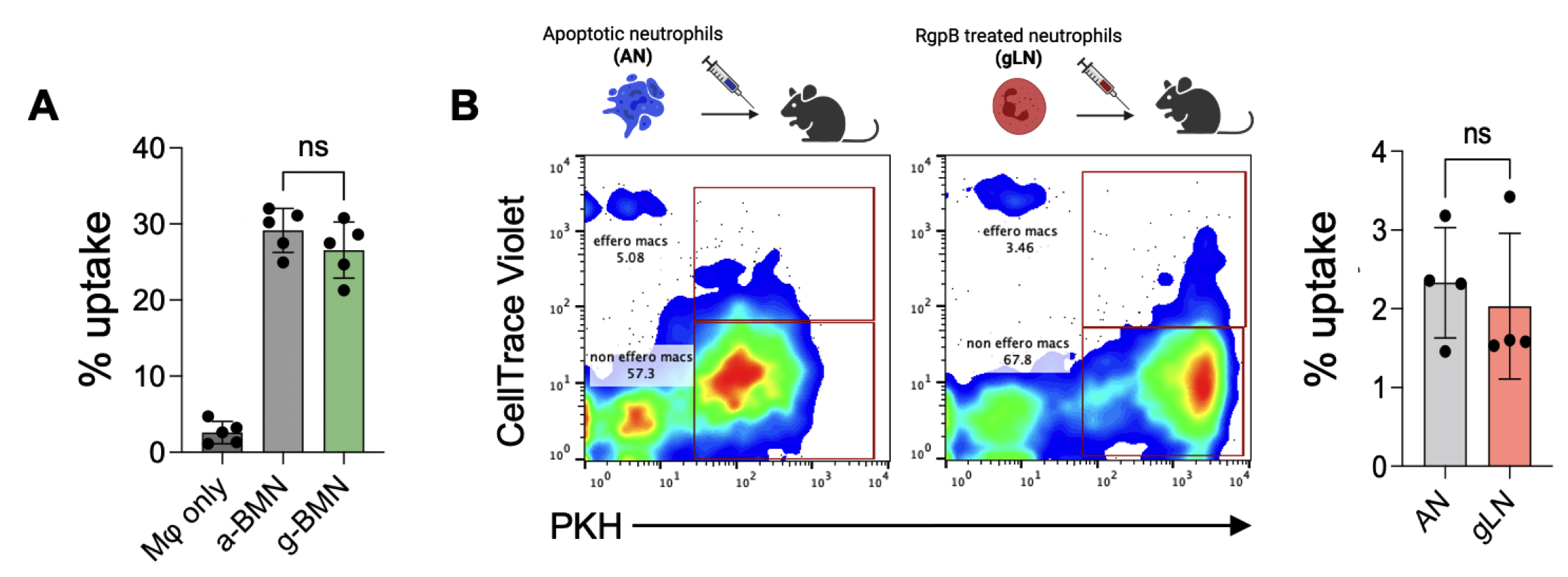

Supplement: Supplementary file 3 — Supplemental Figure S2 [file 41419_2025_7808_MOESM3_ESM.tif]

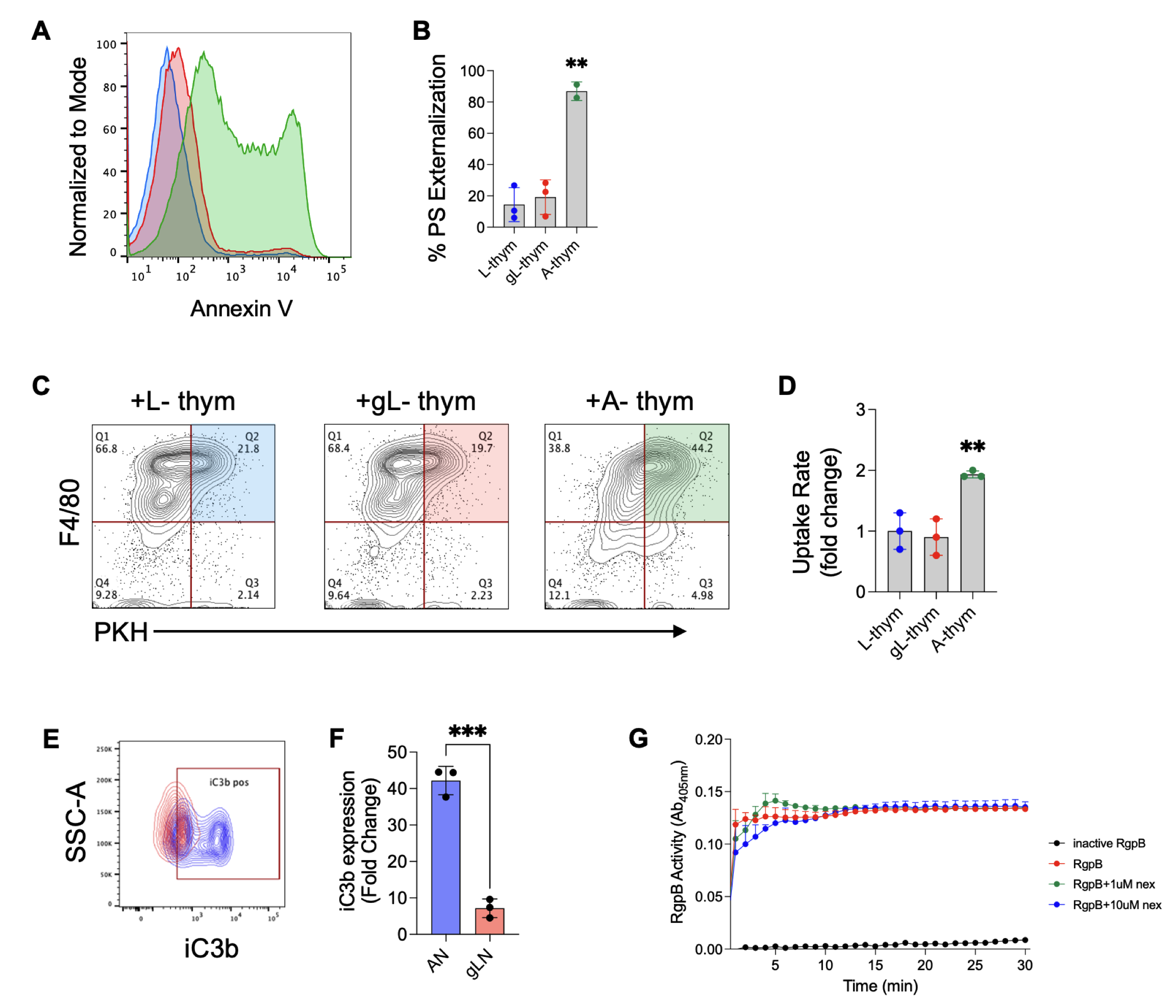

Supplement: Supplementary file 4 — Supplemental Figure S3 [file 41419_2025_7808_MOESM4_ESM.tif]

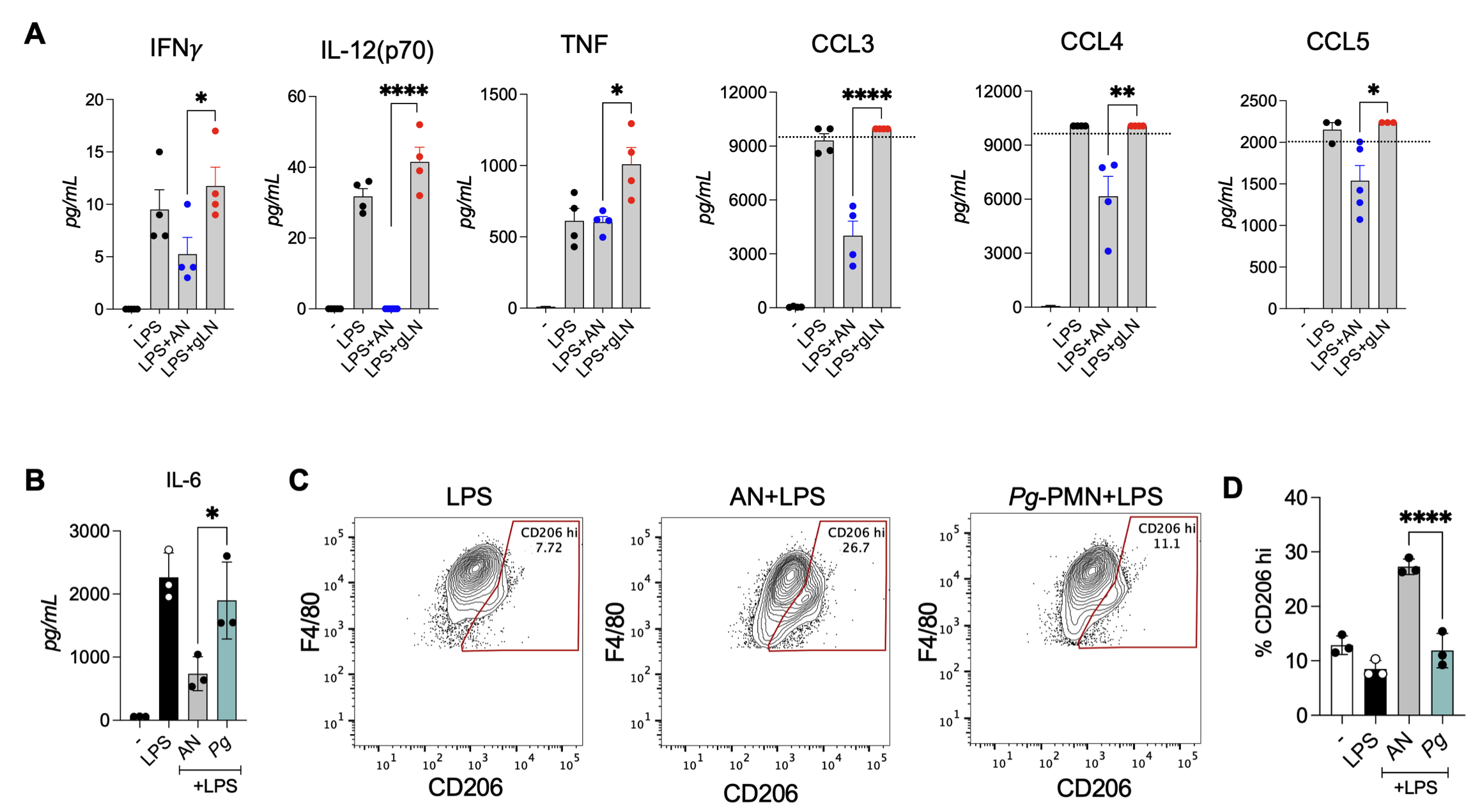

Supplement: Supplementary file 5 — Supplemental Figure S4 [file 41419_2025_7808_MOESM5_ESM.tif]

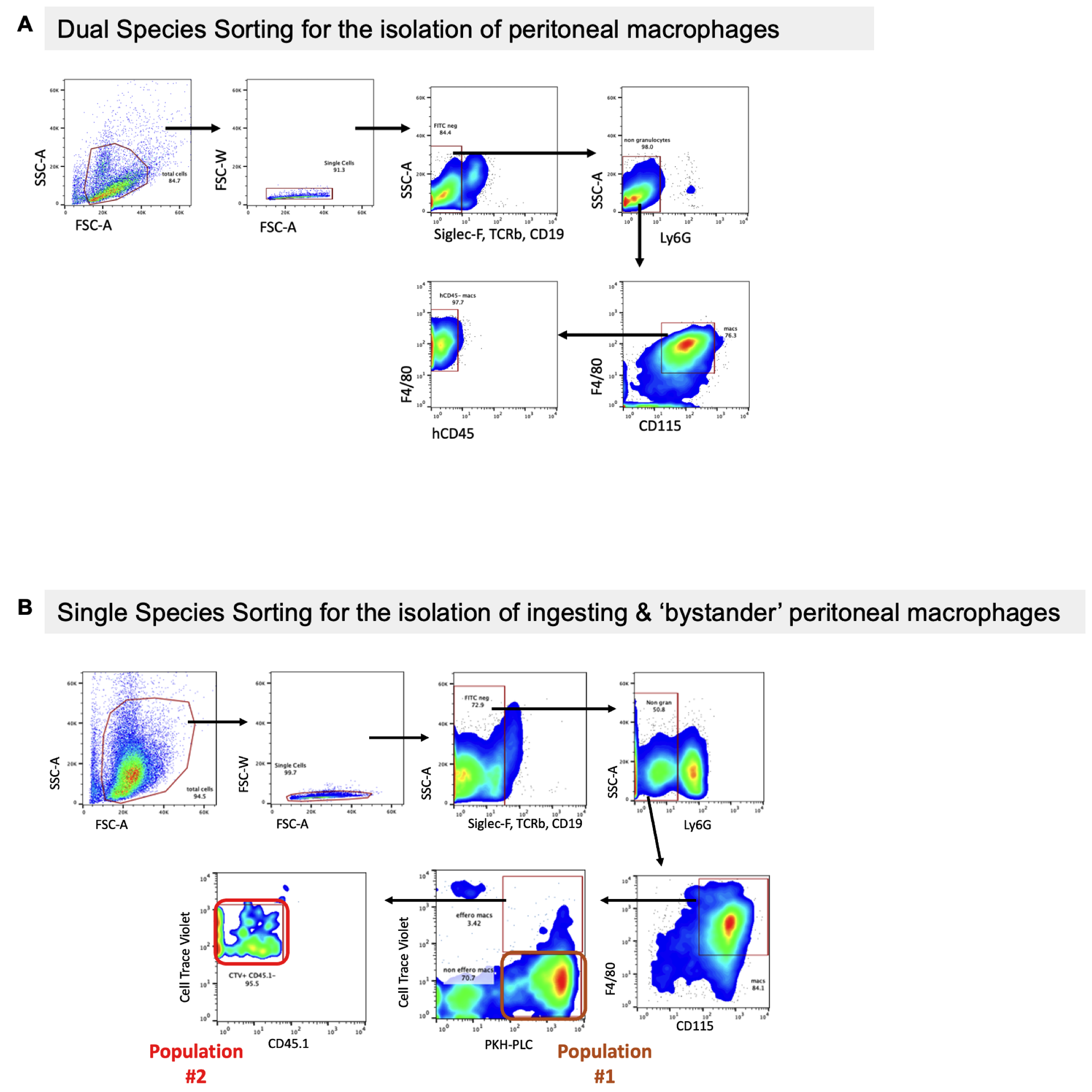

Supplement: Supplementary file 6 — Supplemental Figure S5 [file 41419_2025_7808_MOESM6_ESM.tif]

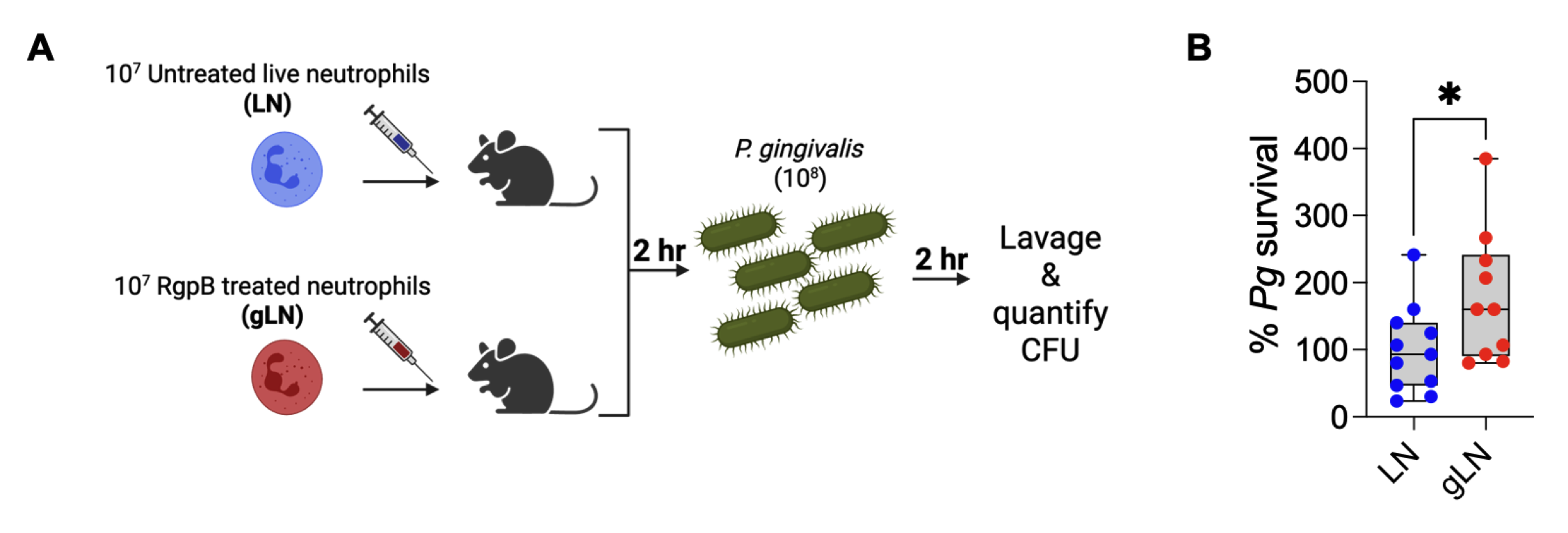

Supplement: Supplementary file 7 — Supplemental Figure S6 [file 41419_2025_7808_MOESM7_ESM.tif]
